# Advances and challenges in cell–cell communication inference: a comprehensive review of tools, resources, and future directions

**DOI:** 10.1093/bib/bbaf280

**Published:** 2025-06-19

**Authors:** Giulia Cesaro, James Shiniti Nagai, Nicolò Gnoato, Alice Chiodi, Gaia Tussardi, Vanessa Klöker, Carmelo Vittorio Musumarra, Ettore Mosca, Ivan G Costa, Barbara Di Camillo, Enrica Calura, Giacomo Baruzzo

**Affiliations:** Department of Information Engineering, University of Padova, Via Gradenigo 6/B, 35131, Padova, Italy; RWTH Aachen Medical Faculty, Institute for Computational Genomics, Pauwelsstrasse 19, 52074, Aachen, Germany; Department of Biology, University of Padova, Via Ugo Bassi 58/B, 35131, Padova, Italy; Institute of Biomedical Technologies, National Research Council (CNR), Via F.lli Cervi 93, 20054, Segrate (Milan), Italy; Department of Information Engineering, University of Padova, Via Gradenigo 6/B, 35131, Padova, Italy; RWTH Aachen Medical Faculty, Institute for Computational Genomics, Pauwelsstrasse 19, 52074, Aachen, Germany; Department of Biology, University of Padova, Via Ugo Bassi 58/B, 35131, Padova, Italy; Institute of Biomedical Technologies, National Research Council (CNR), Via F.lli Cervi 93, 20054, Segrate (Milan), Italy; RWTH Aachen Medical Faculty, Institute for Computational Genomics, Pauwelsstrasse 19, 52074, Aachen, Germany; Department of Information Engineering, University of Padova, Via Ugo Bassi 58/B, 35131, Padova, Italy; Department of Biology, University of Padova, Via Ugo Bassi 58/B, 35131, Padova, Italy; Department of Information Engineering, University of Padova, Via Gradenigo 6/B, 35131, Padova, Italy

**Keywords:** cell–cell communication, cellular signaling, ligand-receptor interaction, single cell transcriptomics, spatial transcriptomics

## Abstract

Recent advancements in high-resolution and high-throughput sequencing technologies have significantly enhanced the study of cell–cell communication inference using single-cell and spatial transcriptomics data. Over the past 6 years, this growing interest has led to the development of more than 100 bioinformatics tools and nearly 50 resources, primarily in the form of ligand-receptor databases. These tools vary widely in their requirements, scoring approaches, ability to infer inter- and/or intra-cellular communication, assumptions, and limitations. Similarly, cell–cell communication resources differ in many aspects, mainly in the number of annotated interactions, species coverage, and their focus on inter-cellular signaling or both inter- and intra-cellular communication. This abundance and diversity create challenges in identifying compatible and suitable tools and resources to meet specific user needs. In this collaborative effort, we aim to provide a comprehensive report on the current state of cell–cell communication analysis derived from single-cell or spatial transcriptomics data. The report reviews existing methods and resources, addressing all relevant aspects from the user’s perspective. It also explores current limitations, pitfalls, and unresolved issues in cell–cell communication inference, offering an aggregated analysis of the existing literature on the topic. Furthermore, we highlight potential future directions in the field and consolidate the collected knowledge into CCC-Catalog (https://sysbiobig.gitlab.io/ccc-catalog), a centralized web platform designed to serve as a hub for bioinformaticians and researchers interested in cell–cell communication inference.

## Introduction

Cell–cell communication (CCC) orchestrates cellular interactions within biological systems and is key to understanding tissue physiopathology. CCC mechanisms range from direct cell–cell contact to signaling via proteins, hormones, neurotransmitters, and other molecules, influenced by drugs, chemicals, and pathogens. A primary level of CCC can be studied through ligand-receptor (LR) interactions, which, upon receptor activation, trigger intracellular signaling cascades that alter cell behavior [1].

Recent advances in single-cell RNA sequencing (scRNA-seq) and Spatial Transcriptomics (ST) have enhanced our ability to characterize cellular heterogeneity and composition [2], enabling data-driven CCC analysis. However, CCC inference from expression data remains challenging, requiring bioinformaticians to navigate through dozens of different bioinformatics tools, each with distinct scoring functions and LR databases, while molecular biologists must understand limitations and assumptions of current tools and resources.

The proposed review aims to offer a comprehensive overview of the field of CCC inference from scRNA-seq and ST data, summarizing state-of-the-art tools and resources, discussing current and future challenges, and ultimately assisting users in the selection of optimal analyses settings.

We also developed CCC-Catalog (https://sysbiobig.gitlab.io/ccc-catalog), an online resource that compiles available CCC resources and methods. The platform enables users to filter and select resources and tools based on their specific needs, facilitating the identification of the right CCC analysis workflow. In addition, the CCC-Catalog brings together current literature, including review articles, comparative studies, and benchmarking analyses, serving as a comprehensive hub for researchers in the field of CCC analysis.

## Ligand-receptor databases and CCC resources

Ligand-receptor databases serve as the foundational ingredient for inferring CCC. However, in the dynamic landscape of CCC studies, the diversity of LR interaction databases introduces significant challenges, such as results heterogeneity and inconsistency among datasets. Supplementary Table S1 and Supplementary Data 1 offers a comprehensive list of 26 LR databases, with detailed descriptions of their content, including the number and types of interactions, the sources used for their creation, the software tools they are associated with, and other key aspects we retained relevant from a user’s perspective. Supplementary Section S1 includes details on the strategies pursued in collecting the LR databases and the main excluded databases.

**Figure 1:**
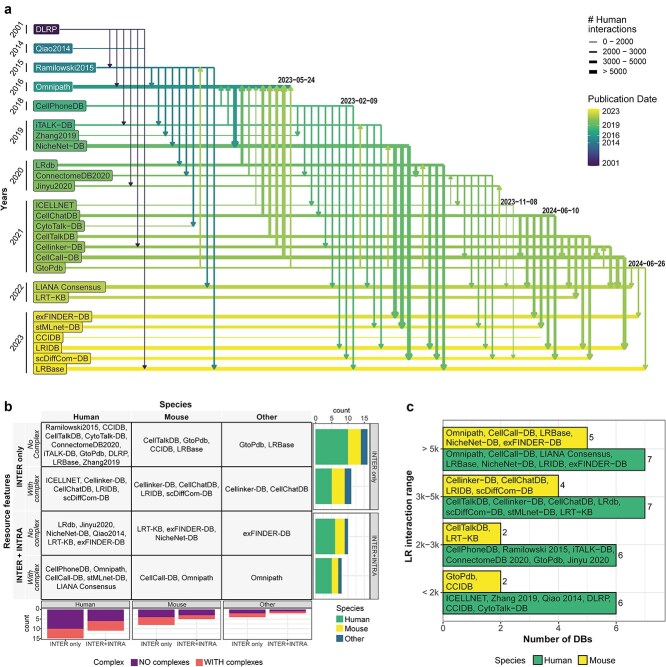
CCC resources. Panel a) ‘the history’ of LR databases and their sources. Databases are ordered by date of publication on the Y-axis and last updates are indicated in the label at the end of each row. Vertical arrow indicates that the database at the starting point has been included in the database at the ending point. Arrows going up indicate an update, after the publication, with the inclusion of the newer source. Panel b) classification of CCC resources based on species (human, mouse and other) and included features (INTER only: Information only on intercellular signaling, i.e. LR pairs, including or omitting information about complexes/subunits; INTER + INTRA: Information about intercellular signaling, including or omitting information about complexes/subunits, and intracellular signaling). Stacked barplots on the right show the amount of CCC resources handling the different species. Stacked barplots on the bottom show the amount of CCC resources handling complexes/subunits, stratified by resources that handle only intercellular signaling and ones that handle also intracellular signaling. Panel c) classification of LR databases based on the number of interactions proposed for human and mouse species.

In recent years, an increasing number of LR databases have emerged from the integration of previous resources or from existing cell signaling databases (Fig. 1a). This process involves careful selection, curation, and refinement aimed at identifying and enhancing the set of LR interactions employed in CCC tools.

The screened LR databases demonstrate remarkable diversity, not only in the number of annotated LR pairs, spanning from a few hundred to several thousand, but also in LR interaction specificity and in the types of communication (i.e. intercellular signaling only versus both inter- and intra-cellular signaling) (Fig. 1b and 1c). Most databases are based on human or mouse interactions [3–6], with few focused on other model organisms or plants [7, 8]. The LR databases may vary in being generic or associated with specific tissues or biological topics (e.g. [9] for hematopoietic stem cells and [10] for neural signaling). Although some databases are highly specific to certain species or tissues, Dimitrov et al. showed limited uniqueness across resources, with most sharing a substantial portion of their core data, including interactions, transmitters, and receivers [11]. Additionally, LR databases are designed to consider only inter-cellular communications [3, 12, 13] or both intercellular and intracellular LR interactions [11, 14, 15]. The interaction type can be one-to-one or account for protein complexes, recognizing that multiple proteins are required for downstream signal activation [5, 12, 16]. In this scenario, CellPhoneDB and OmniPath are widely used because they integrate a broad spectrum of data from various species and tissue types.

The evidence emerging from high-throughput studies of protein–protein interactions shows that interactions, even more than singular proteins, vary across tissues, cell types, functions and diseases [17]. Moreover, pathway databases often exhibit representation biases toward specific signaling pathways or functional categories [18]. This highlights that not all resources are equally comprehensive or reliable in representing the cell connectome. In this context, characterized by biological variability and knowledge-bias, well-curated resources that allow users to select specific types of interactions are strongly needed. However, the choice of LR interactions is a debated issue that requires careful consideration, and no consensus exists on evaluating them. Some databases provide meticulous curation, with qualified experts carefully assessing and selecting CCC interactions from available data. Others provide citations or refer to experimental evidence supporting interactions.

On one hand, choosing a highly curated and focused resource can reduce false positives but may limit the identification of all relevant interactions, i.e., increasing false negatives. On the other hand, selecting a broader curated resource increases the risk of false positives, potentially compromising the reliability of CCC analysis results. The selection of a LR database is a critical trade-off between database comprehensiveness and the potential risk of misclassification errors in inferred interactions, which underscores the importance of strategically aligning the choice of LR database with the specific goals of each study. Thus, curating the inclusion of specific protein interactions or tissue-specific data that are expected to be found in the samples under analysis and in the same way excluding LR interactions that are too rare or completely unexpected is highly suggested. An informed choice ensures that insights derived from CCC studies are accurate, relevant, and current—reflecting both the true complexity of cellular interactions and the evolving completeness of biological knowledge. Finally, recent advances in powerful artificial intelligence (AI)-based tools [19, 20] have enabled large-scale prediction of protein structures and interactions, including LR complexes. The integration of such approaches is likely to become a standard feature in LR databases CCC in the near future. These strategies have the potential to bridge gaps in biological knowledge and provide unprecedented access to molecular-level information. However, they also introduce a degree of uncertainty, as their predictions are computational inferences rather than experimentally validated facts. Therefore, critical evaluation, clear labeling of predicted data, and greater transparency in database construction are—and will remain—essential to ensure the informed and reliable use of LR datasets.

## C‌CC methods

**Figure 2:**
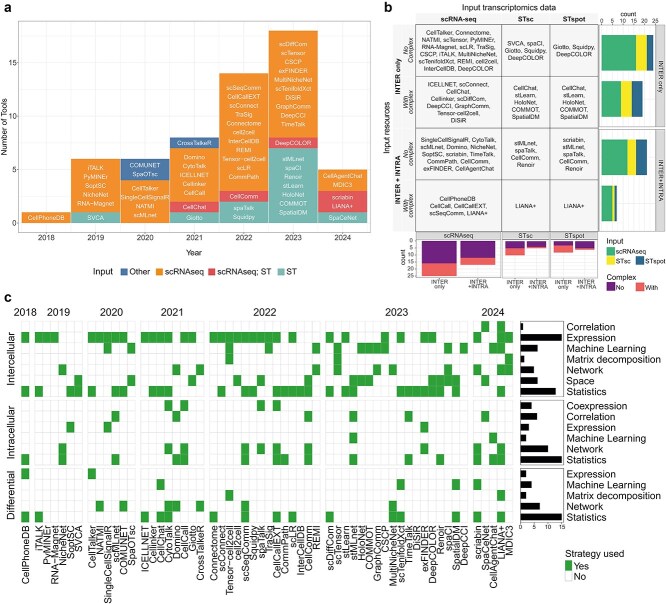
CCC tools. Panel a) distribution of CCC tools published each year, categorized by the type of input data utilized. The year 2022 marked a notable increase in the number of CCC tools, with the introduction of tools capable of processing both scRNA-seq and spatial transcriptomic (ST) data. While scRNA-seq remains the dominant input data type, the number of tools tailored for ST is steadily growing. A small subset of tools utilizes alternative data types, categorized as ‘other’. The year reported in figure is the year of the first publication of each tool. This analysis includes tools published up to July 2024. Panel b) classification of CCC tools based on input transcriptomics data (scRNA-seq, ST with single-cell resolution [STsc], or ST with spot resolution [STspot]) and input resources (INTER only: A priori information on intercellular signaling, including or omitting information about complexes/subunits; INTER + INTRA: A priori information about intercellular signaling, including or omitting information about complexes/subunits, and intracellular signaling). Stacked barplots on the right show the amount of CCC methods handling the different types of input transcriptomics data. Stacked barplots on the bottom show the amount of CCC methods handling complexes/subunits in LR resources, stratified by methods that handle only intercellular signaling and ones that handle also intracellular signaling. Panel c) strategies employed by CCC tools for intercellular, intracellular, and differential score calculations. Tools are grouped by year to illustrate trends in the adoption of specific methodologies over time. A barplot highlights the frequency of each methodology’s usage. A detailed taxonomy and explanation of the implemented methodologies are provided in the Supplementary Section S3. Features correspond to the latest tool version; publication year refers to the tool’s initial release.

In recent years, the number of methods for CCC analysis has grown significantly (Fig. 2a), reaching around 100 tools by July 2024 (Supplementary Data 2 and Supplementary Section S2).

Available methods differ in numerous aspects (Supplementary Fig. S1), some of which are more evident and directly impact method selection (Fig. 2b). For example, the type of input data, with some methods only accepting scRNA-seq data or ST data, determines whether a method can be used in a given analysis. Similarly, the type of inference (e.g. intercellular only versus intercellular+intracellular, differential, time series analysis) or the resolution at which prior information is used (e.g. considering subunits in LR databases) also directly influences method choice and analysis results.

Less immediate but still impactful aspects include implementation choices (e.g. programming language, parallelization) that determine the dataset size that can be analyzed and/or the computational resources required. Additionally, the way CCC is measured, whether prioritizing specificity or intensity, also plays a role. Other aspects are of greater interest to method developers, such as the methodologies employed (e.g. statistical-based, network-based, ML-based) and how they can be combined to achieve more robust and accurate CCC inference.

This section analyzes the key features of CCC methods: the transcriptomic data type they accept as input (Section 3.1), whether and how they identify intercellular (Section 3.2) and intracellular (Section 3.3) communication, or their alteration under different experimental conditions (Section 3.4), the output type and meaning, including visualizations (Section 3.5), implementation details (Section 3.6), and main application fields (Section 3.7).

Where relevant to the discussion, we will also classify the methods based on the methodologies used: network-based, statistics-based, correlation-based, coexpression-based, expression-based, ML-based, space-based and matrix decomposition-based methods. Complete definitions of this classification are in Supplementary Section S3.

This review discusses a broad subset of 58 methods for CCC analysis, covering various approaches (Supplementary Data 2). Supplementary Section S2 describes the strategies employed for tool collection, as well as the inclusion and exclusion criteria.

### Methods’ inputs

#### Gene expression data

State-of-the-art methods for CCC inference have increasingly focused on analyzing scRNA-seq and ST data. While most methods accept only scRNA-seq data as input, an increasing number of tools handle both scRNAseq and ST data, while some methods are specific for only ST data (Supplementary Table S2 and Supplementary Data 2).

Among the 58 tools reviewed, 43 can analyze scRNA-seq, with 32 exclusively designed for this data type (Fig. 2a-b, Supplementary Fig. S1). Conversely, five tools - CellChat, Scriabin [21], CellComm [22], LIANA+ [23], and DeepCOLOR [24] – also support ST data. A unique case is SpaOTsc [25], that requires both scRNA-seq and single-cell resolution ST data, since the model estimates the CCC likelihood by employing optimal transport to find the optimal cell mapping from scRNA-seq data to their spatial positions, based on ST data. Other six tools - NATMI, ICELLNET [16], Tensor-cell2cell [26], cell2cell [27], NicheNet, and REMI [28] - are also compatible with bulk RNA-seq data.

A total of 12 tools have been specifically developed for ST data, most supporting both single-cell resolution data (e.g. MERFISH [29], Seq-Scope [30], Stereo-seq [31]) and spot-based resolution data (e.g. 10X Visium [32], Slide-Seq [33], GeoMX [34]). These versatile tools include Giotto [35], stLearn [36], Squidpy [37], stMLnet [38], HoloNet [39], spaTalk [40], COMMOT [41], Renoir [42], and SpatialDM [43], while SVCA [44], SpaCeNet [45], and spaCI [46] focus exclusively on single-cell resolution ST data.

Additionally, tools that use CCC inference results as input for downstream analyses have also emerged, namely COMUNET [47] and CrossTalkeR [48].

While transcriptomics data and LR databases are common inputs for all methods, some methods require or accept additional data. For example, InterCellDB [49] requires differentially expressed genes to compute intercellular signaling scores. TraSig [50], which is mainly designed for studying CCC in developmental biology, needs precomputed pseudotime trajectories.

Other tools (CellPhoneDB v4.1 [51], NicheNet [15], COMUNET, SoptSC [52], TraSig, GraphComm [53], CellAgentChat [54], MDIC3 [55]), accept optional input to customize or enhance CCC analysis for specific needs. This additional input typically includes lists of genes of interest or gene-regulatory networks.

#### LR databases and CCC resources

CCC inference tools can be broadly categorized into three types based on how they handle LR databases: built-in databases, user-specified databases and database agnostic (Supplementary Table S2 and Supplementary Data 2). Tools with built-in databases, such as CellChat and CellPhoneDB, offer a streamlined approach by using pre-loaded, curated and developer-validated knowledge bases. This can simplify the user experience and ensure consistency in predictions. However, these built-in resources may not always align with the specific requirements of each research context. In contrast, user-specified tools like NATMI and ICELLNET provide greater flexibility by enabling users to incorporate their own prior knowledge resources. This adaptability is advantageous for studies involving uncommon species, specific tissues or experimental conditions.

As previously discussed, the choice of CCC resources is critical, as the quality and comprehensiveness of annotated interactions can deeply influence the achieved results. On the other hand, some recent approaches perform CCC inference without the use of a LR database. These methods often leverage other aspects of the data, such as gene regulatory networks or spatial coordinates from ST. Examples of database-agnostic tools are MDIC3, SpaCeNet, and SpaOTsc.

#### Intercellular communication analysis

Transcriptomic-derived intercellular CCC often relies on two primary inputs: a gene expression matrix and a LR database. With different flavors, CCC inference tools identify interactions as tuples (source, target, ligand, receptor, score), where ‘*score*’ refers to an ‘intercellular score’, i.e. a measure of evidence of intercellular signaling. Despite a shared overall structure, CCC inference tools vary significantly in the methodologies used to compute intercellular scores, affecting both the nature and interpretation of the resulting scores. These methodologies range from expression-based, correlation-based, and statistics-based approaches to more complex models such as network-based, machine learning (ML)-based, matrix decomposition-based and space-based methods, as well as hybrid approaches. Over the years, there has been a clear shift toward ML and network-based approaches, with increasing development of tools focused on ST data (Fig. 2c).

The scoring methodology significantly shapes the interpretation of intercellular communication, with each approach offering distinct advantages and limitations. CCC inference methods can produce either binary or continuous scores (Supplementary Table S3 and Supplementary Data 2). *Binary scoring*, employed by tools like CellTalker [56], iTALK [13], and scMLnet [14], assumes a high expression threshold for detecting communication, making it straightforward to implement but lacking in results prioritization, making experimental validation difficult. *Continuous scoring*, on the other hand, offers a more refined quantitative assessment, enabling nuanced insights and prioritization for further research.

Scoring methods for LR pairs can focus on specificity or intensity (Supplementary Table S3 and Supplementary Data 2). *Specificity-focused scoring*, used by tools like CellPhoneDB [57] and CellChat [12], emphasizes unique or cell-type-specific interactions. This approach, often supported by statistical tests like permutation analysis, identifies rare signaling events but may miss the broader significance of more common interactions. *Intensity-focused scoring*, on the other hand, evaluates the strength of interactions, capturing high-activity signaling but potentially overlooking biologically important low-intensity interactions. Several tools use both scoring schemes, prioritizing both unique interaction mechanisms and interaction strength.

Moreover, multi-subunit protein complexes present another layer of complexity in CCC analysis (Supplementary Table S3 and Supplementary Data 2). Efremova et al. first addressed this by proposing the use of minimum expression values for genes within protein complexes. Our review found that 33% of CCC tools consider multimeric receptors in their analyses, with an increasing number of such tools in recent years.

Advances in CCC analysis include approaches like Scriabin [21], which cluster cells based on their intercellular interaction programs to reveal communication niches through transcriptomics. Latest tools, such as COMMOT [41], DeepColor [24], and DeepCCI [58], utilize both cutting-edge methodologies to enhance the precision and scope of CCC inference (e.g. generative modeling, and deep learning), and spatially resolved transcriptomics (unimodal/multimodal).

### Intracellular communication analysis

While most CCC tools focus on inferring LR interactions, a significant percentage (36%) also model downstream intracellular signaling (Fig. 2b-c, Supplementary Fig. S1, Supplementary Table S4 and Supplementary Data 2). These methods aim to computationally model the intracellular processes triggered within the receiving cell type by LR interactions, providing a more comprehensive view of CCC and reducing false positives. Unlike intercellular methods, intracellular scoring schemes exhibit significant diversification in the biological processes they model.

Intracellular tools can be classified as *resource-independent* and *resource-dependent* tools based on their reliance on external resources (Supplementary Table S4 and Supplementary Data 2). The first class, such as CytoTalk [59], TimeTalk [60], and SpaCeNet, constructs intracellular networks de novo using data-driven approaches, such as co-expression analysis or probabilistic graphical models. These methods are flexible and can capture novel interactions, but their reliance on data quality increases the risk of false positives. In contrast, resources-dependent tools rely on established signaling pathways or transcriptional regulatory network databases. While offering high reliability, they are constrained by the accuracy and curation of existing resources, potentially missing novel interactions.

Independent of the resources used, the tools employ a range of distinct strategies, often combining them (e.g. statistics-based, expression-based, co-expression-based, correlation-based, network-based, and ML-based). (Fig. 2c and Supplementary Data 2). Examples of statistics-based tools include CellPhoneDB [51], CommPath [61], and Renoir, while expression-based tools include SingleCellSignalR [62] and SoptSC [52].

Network propagation algorithms, such as Random Walk, are commonly used (e.g. scSeqComm [63], NicheNet, spaTalk, Scriabin), alongside other methods like shortest/causal path [23, 64] or examining signal flows in intracellular networks/pathways [22, 65]. stMLnet, which uses a Random Forest regressor to determine the importance score of each LR pair contributing to the target gene expression, and CellAgentChat, which uses a neural network regressor to predict gene expression in a cell, were classified as ML-based.

Most tools primarily focus on intracellular signaling targeting transcriptional regulatory activity as an effect of LR binding, typically modelling intercellular and intracellular signaling separately, and then linking the receptor to the transcription factor activity [14, 22, 23, 40, 51, 63, 66]. In contrast, some tools incorporate LR pairs into their models, associating ligands with target genes [15, 42, 65] or linking LR activity to transcriptional activity [54, 60].

The outputs of these tools can be both *quantitative* and *qualitative* (Supplementary Table S4 and Supplementary Data 2). Quantitative outputs typically measure intracellular signaling activation by assessing transcriptional intensity or downstream gene impacts, while qualitative outputs infer signaling networks, providing a conceptual framework of molecular interactions and relationships.

The diversity of computational methods reflects the complexity of intracellular processes and their critical role in shaping cellular functions and response.

### Differential cellular communication analysis

Studying different biological conditions (e.g. diseased versus control) helps identify factors driving distinct phenotypes. Genetic, temporal, or pathological states influence cellular behavior and interactions [67, 68], thus understanding differential communication across conditions provides key insights into underlying mechanisms.

About 38% of the reviewed tools implement differential CCC analysis, and they can be categorized into two main types (Fig. 2c, Supplementary Fig. S1, Supplementary Table S5 and Supplementary Data 2) based on how they account for intra-condition variability, when present (e.g. multiple samples/subjects within each experimental condition). Most methods disregard intra-condition variability, even when available, by aggregating all cells from different samples within the same condition and focusing solely on overall condition-level differences. However, few tools incorporate the variability by considering the single samples during the inference process to refine condition-level analysis. These tools are particularly valuable for studies with large cohorts or significant sample heterogeneity, where intra-condition variability plays a crucial role.

Most tools rely on pairwise comparisons of CCC, using different computational strategies (Fig. 2c and Supplementary Data 2). These range from simple intersections of condition-specific signaling events (e.g. CellTalker and Domino [66]), to statistical approaches, such as differential analysis of ligands and receptors (e.g. CellPhoneDB [69], CellChat [70], iTALK, multiNicheNet [71], LIANA+) and intercellular or distance metrics (e.g. ICELLNET [72], scDiffCom [73], scSeqComm, scLR [74], scTenifoldXct [75], SpatialDM ). Some tools, like COMUNET and CrossTalkeR, leverage intercellular interactions predicted by other tools to build network of differentially interacting cell types. Few tools also focus on comparing intracellular signaling by using network analysis to detect altered downstream signaling, inferring altered intracellular network (e.g. CytoTalk, Domino, LIANA+) or providing quantitative metrics of such alteration (e.g. scSeqComm, NicheNet).

Additionally, dimensionality reduction methods like matrix decomposition-based (e.g. Tensor-cell2cell, LIANA+) and ML-based approaches (e.g. CellChat, Scriabin) enable the simultaneous comparison of more than two conditions, allowing researchers to identify broader communication patterns (Supplementary Table S5 and Supplementary Data 2).

### Output, including visualization

Among current CCC inference tools, five principal categories of tool output can be identified: scores, network, list of active genes or LR, tensor decomposition results, and cell-level information (Supplementary Table S6 and Supplementary Data 2).

Output scores are produced by 45 tools, providing information on specificity, intensity, or significance of inferred interactions, depending on the scoring strategy implemented (Section 3.2). Other 11 tools output interaction networks including information on cell-types, LRs pairs, or transcription factors and target genes. 4 tools (CellTalker, iTALK, COMUNET, and MultiNicheNet) return lists of genes involved in the communication, typically LR pairs. Only two tools (scTensor [76] and Tensor-cell2cell) implement tensor decomposition and return the results of this algorithm (e.g. tensor loadings). Different from all previous tools, TraSig and CSCP [77] provide as output information on cells and sub-groups of cells having similar communication patterns. One tool (LIANA+) outputs tensor decomposition results along with intercellular communication scores and a list of genes associated with intracellular signaling pathways.

The type of output significantly affects subsequent analyses. For example, tensor decomposition loadings and factors are difficult to interpret, complicating the biological interpretation. Conversely, lists of LR pairs provide straightforward biological insights, but the absence of a score prevents results prioritization and design of experimental validation. Numerical scores provide users with remarkable flexibility for conducting subsequent analyses and setting custom thresholds for communication results. Nevertheless, accurately interpreting the biological significance of numerical scoring outputs requires the comprehension of the mathematical formulations used by each method.

Given the extensive output of some packages, visualization is essential for interpreting CCC analysis results helping researchers in identifying and prioritizing key insights (Supplementary Table S6 and Supplementary Data 2). Common visualizations include bar plots, heatmaps, and spatial plots, which display top-scoring results alongside statistical significance and spatial context. Dot plots and violin/box plots are often used to combine score ranges with their distribution and density. For inferred communications, popular methods include circos plots, network plots, and Sankey plots. Most packages offer multiple visualization options, with CellChat standing out by providing six types: circos plots, dot plots, heatmaps, network plots, Sankey plots, and spatial plots. Notably, seven tools (CellPhoneDB, CSCP, RNA-magnet [78], spaCI, spaOTsc, SpaCeNet and TimeTalk) lack visualization functions. The complexity and diversity of CCC results make visualization challenging. To address specific visualization needs, some tools (e.g. scConnect [79], CellChat [70], Giotto [35], and CellAgentChat [54]) offer interactive WebApps, simplifying result exploration and enabling non-bioinformaticians to investigate outputs effectively.

### Software

R and Python are undoubtedly the most widely used programming languages in the CCC community (Supplementary Fig. S2, Supplementary Table S7 and Supplementary Data 2). Some CCC tools leverage both, using Python for computation and R for data preprocessing or visualization. Additionally, many R packages integrate C++ or Matlab to improve computational performance. While R dominated the early years of CCC research due to its rich ecosystem, there is a growing shift toward Python, reflecting broader trends in bioinformatics.

The usability, reproducibility, and adoption of CCC tools heavily depend on their quality and release practices. Tools with comprehensive documentation, test datasets, installation guides, and compatibility with popular bioinformatics packages significantly enhance the user experience. Packaging tools as Python libraries or R packages, rather than standalone scripts, ensures better modularity and easier integration into existing workflows, driving progress in CCC studies.

Overall, CCC software is of high quality: most of the reviewed tools are structured as R or Python packages and typically include installation instructions, tutorials, and test datasets (Supplementary Table S7 and Supplementary Data 2).

### Applications and case studies

CCC inference has been successfully applied in various research fields, from disease and infection research to physiological processes. As a representative sample, we reviewed the CCC case-studies described in the 58 CCC tool manuscripts included in this study (Supplementary Data 2).

Humans and mice are the most studied species. Due to ethical constraints and limited sample availability in human research, mouse models serve as essential tools for genetic manipulation and controlled experiments.

Biological applications demonstrate disparate trends across species and research fields (Supplementary Fig. S3). In mice, CCC studies predominantly focus on the brain and nervous system (25 out of 52 case studies), followed by embryogenesis (12 studies), emphasizing CCC analysis relevance in developmental biology. In human research, cancer is the most studied field, with 29 of 72 case studies exploring CCC dynamics in tumors. Other key areas include embryogenesis (6 studies) and viral diseases (9 studies). As sequencing techniques and computational methods advance, CCC applications will likely expand, providing deeper biological insights and novel therapeutic strategies.

## Current pitfalls and open issues on cell–cell communication analysis

### Validation: the elephant in the room

The primary open issue in CCC analysis is the absence of a definitive ground truth for validating methods performance. As CCC inference is data-driven, accuracy depends on the computational approach used [23]. To address this, researchers employ various validation strategies (Supplementary Table S8 and Supplementary Data 2).

One popular validation method is *literature agreement*, i.e. cross-referencing predictions with existing biological literature, but it fails to capture yet to be discovered interactions. *Experimental validation* includes (i) confirming protein expression with immunohistochemistry and protein staining; (ii) verifying spatial localization of communicating cells through fluorescence in situ hybridization and flow cytometry; (iii) assessing the functional role of CCC mediators through perturbation experiments (e.g. knockout studies or Perturb-seq experiments). However, this approach is often limited to a few LR pairs and few cell types per experiment. *Indirect validation* using data generated from other emerging technologies, such as spatial transcriptomics, proteomics or bulk RNA-seq data, is also very used. Particularly, spatial colocalization validation assumes that spatially adjacent cell types are more likely to communicate, supporting predicted interactions with indirect evidence. Methods for experimental and indirect validation have been thoroughly reviewed by Armingol et al. [80]. However, two techniques from the field of spatial transcriptomics stand out as particularly valuable for enhancing and validating CCC tools. The first one, Visium HD [81], achieves single-cell scale resolution and improves tissue analysis, identifying distinct cell subpopulations and cell-to-cell relationships. The second one, multiplexed error-robust FISH (MERFISH) method [82] extends single molecule FISH (smFISH) achieving higher throughput and nanometer-scale resolution, making it ideal for exploring gene regulatory networks and intracellular processes relevant to intercellular communication. *Synthetic dataset validation* uses the ground truth contained in simulated datasets, particularly used for ST-based CCC tools. While simulated datasets offer controlled conditions to test CCC tools, they often fail to replicate the full complexity of CCC or include realistic spatial details. Finally, *robustness evaluation* of the tools is essential to minimize the false positives and negatives and ensure models are resilient to noise or error in the data (e.g. batch effects, dropout events, errors in cell cluster annotations).

Combining multiple evaluation methods is typically suggested, as it provides a more comprehensive evaluation of CCC tools. However, there is an urgent need for a ‘gold standard’ dataset to better assess tool performance, standardize evaluations, and improve the accuracy of CCC tools.

### Software challenges: computational burden and data visualization

The growing size of transcriptomics datasets, including cell atlases with millions of cells, poses major computational challenges for bioinformatics analyses, including CCC inference. Despite this, only 24% of reviewed tools assess computational performance (e.g. runtime, memory usage), and less than half utilize parallel computation (Supplementary Tables S7-S8 and Supplementary Data 2).

Modern gene expression matrices often require dozens of gigabytes of RAM, and limitations in programming languages (e.g. 32-bit indexing in R) can prevent their loading, even with powerful hardware. While compressed or sparse matrix formats reduce memory usage, they can increase computational overhead.

Additionally, R and Python have limited support for shared-memory parallelism (e.g. multithreading), restricting the efficient use of multi-core CPUs in laptops or workstations. Multiprocessing approaches, though effective, often demand data redundancy across processes, increasing memory requirements.

To handle expanding dataset sizes and computational demands, future CCC tools must combine efficient memory management, scalable parallel libraries, and performance-optimized code (e.g. C++ modules). These strategies are essential for maintaining scalability and efficiency in modern bioinformatics workflows.

Interpreting CCC results is a major challenge due to their multidimensional nature, encompassing signaling molecules, sender and receiver cell clusters, computed scores, etc. Interactive inspection and visualization tools have proven invaluable for navigating this complexity. Interfaces like InterCellar, CClens, CellLinker, and stLearn facilitate CCC analysis by providing user-friendly platforms to visualize results from various CCC tools. As the number of CCC tools grows, the availability of dashboards and apps capable of integrating outputs and offering effective and interactive visualizations is increasingly important. Separating result computation from visualization further enhances flexibility, allowing users to mix and match analytical methods with their preferred visualization strategies.

### Trends from available review, comparison and benchmarking studies

The existence of multiple and very different CCC inference tools, the lack of an established and ultimate ground truth to validate CCC tools outputs, and the heterogeneity of CCC resources/databases available, presents significant challenges for benchmarking and evaluating existing CCC analysis workflows. Recently, several initiatives have aimed to address these challenges in the form of review studies (describing available methods and resources), comparison studies (measuring the agreement of different CCC resources or tools predictions) and benchmarking studies (ranking CCC tools based on some metrics).

#### Review studies and comparison studies

The growing landscape of CCC bioinformatics analyses has been explored in nine recent review studies (Supplementary Section S4). These reviews typically categorize CCC tools based on their computational approaches. For example, Shao et al. (2020) and Armingol et al. (2021, 2024) [1, 80, 83] discuss LR interaction-based strategies, with the latter classifying tools into general-purpose frameworks, specialized applications, and hybrid models.

While all review studies account for CCC tools, they often overlook CCC resources, despite the critical role of well-curated LR pairs in accurate inference. Only a few reviews, such as Ma et al. (2021), Armingol et al. (2021), and Peng et al. (2022) [1, 84, 85], thoroughly assess LR databases, focusing on annotation depth, data sources, and completeness. Others, like Jin et al. (2022) [86], overlook this entirely, underscoring a significant gap in the literature. This oversight is critical, as CCC analysis depends heavily on the quality of its databases.

CCC tools’ reviews often focus on specific applications, particularly cancer, where tools are used to study CCC in tumor microenvironments, immunosuppressive signaling, and drug resistance [83–85, 87, 88]. CCC tools have been reviewed in their application to skin biology [86] and broader contexts like inflammation and immune regulation [83, 84]. While real-world applications offer valuable insights into the practical use of CCC tools, these reviews fail to recommend the best-performing tools for specific scenarios.

A recurring challenge highlighted by all these review studies is the validation of CCC tool performance, due to the lack of standardized assessment frameworks. Experimental confirmation for computational predictions is essential, particularly in intricate biological systems like cancer [1, 85, 87, 88]. Experimental validation methods, including proteomics and immunohistochemistry, are outlined in reviews like Peng et al. (2022) and Wang et al. (2023) [85, 88], alongside functional validation through in vitro/in vivo studies. Indirect validations (e.g. enrichment analyses, use of spatial colocalization) show promise but are inconsistently applied, complicating tool comparisons. Literature agreement validation, while extremely common, depends heavily on the availability and quality of prior research [85, 88].

Building on these foundational reviews, two comparative studies further dissect the nuances of tool performance and resource variability (Supplementary Section S4). The LIANA platform [11] systematically compared seven CCC methods and sixteen CCC resources, uncovering a high similarity in included molecules but significant differences in LR pair interactions. This bias, often pathway-specific, underscores the role of CCC resource selection in shaping CCC analyses. LIANA also highlighted the low overlap in top-ranked interactions across tools, attributing this to their diverse scoring strategies. Despite this variability, methods like CellChat, CellPhoneDB, and SingleCellSignalR showed good robustness to noisy data and errors in input resources. Similarly, Wang et al. (2022) [89] compared nine CCC tools for their ability to infer LR interactions and construct CCC networks. Their findings reinforced the heterogeneity in CCC predictions, driven by differences in database reliance, algorithmic filtering, and prediction strategies. Tools like iTALK and NATMI showed greater consistency with curated databases, while methods like CellChat prioritized fewer interactions to reduce noise. Computational performance also varied considerably among tools. These comparison studies highlight the trade-offs in computational efficiency and sensitivity across tools, underscoring the value of employing multiple methods to achieve reliable CCC predictions.

#### Benchmarking studies

In the literature, four key benchmarking studies evaluate the performance of current CCC tools.

- BENCH1: The ESICCC framework, introduced by Luo et al., [90] provides a systematic approach to evaluate CCC inference methods, benchmarking 18 LR inference tools and five LR-target tools. It is the most extensive and comprehensive study in terms of the number of tools analyzed, tested aspects (accuracy, robustness and usability), and scenarios considered.
- BENCH2: Liu et al. [91] study analyzed 15 tools in terms of predicted CCC concordance and accuracy, using both real and simulated dataset.
- BENCH3: Zhang et al. [92] work evaluated 14 tools based on their robustness to various sources of variability and noise, such as data variability (e.g. batch effects), transcriptomic noise (e.g. dropout events), and noise in prior knowledge (e.g. errors in cell cluster annotations). The authors assess robustness both against individual sources of variability/noise, as well as providing an overall aggregated robustness metric.
- BENCH4: Xie et al. [93] analyzes eight scoring schemes in terms of prediction accuracy, using curated scRNA-seq data from idiopathic pulmonary fibrosis.

Detailed summaries of the four benchmarking studies can be found in Supplementary Sections S4 and S5.

**Figure 3:**
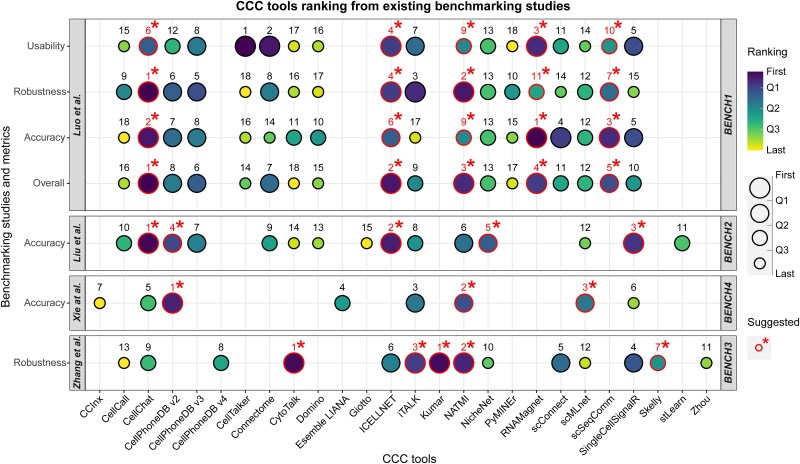
Aggregated analysis of benchmarking studies for CCC tools. This figure presents an aggregated analysis of four benchmarking studies evaluating CCC tools based on available metrics: Accuracy, robustness, usability, and overall performance. Each study’s results are visualized using circles, where the circle’s diameter and color encode the ranking of each tool for a given metric. The numbers close to the circles indicate the ranking position. Tools identified by the study authors as the best-performing are highlighted with red circles, numbers and asterisk (‘*’). Additional details about the data and methodology used in this figure are provided in Supplementary Section S5.

Although these benchmarking studies use different criteria/metrics to assess tools’ performance, and often evaluate different aspects (e.g. accuracy, robustness, or usability), Fig. 3 attempts to summarize the results of these studies by highlighting the top-performing tools, as well as their ranking. Details on the individual studies and how Fig. 3 was constructed are reported in Supplementary Section S5.

Tools’ accuracy has been evaluated in BENCH1, BENCH2 and BENCH4. CellChat is identified as one of the top methods in BENCH1-BENCH2, but holds a mid-to-low position in the ranking of BENCH4. Similarly, SingleCellSignalR ranks well in BENCH1-BENCH2, but is second to last in the BENCH4. CellPhoneDBv2 is ranked first in BENCH4, among the top performers in BENCH2, and slightly above mid-table in BENCH1. iTALK holds a mid-high position in BENCH4, mid-table in BENCH2, and is among the worst performers in BENCH1. NATMI is highly ranked in BENCH4, and around mid-table in BENCH1-BENCH2. scMLnet has a mid-high position in BENCH4, but ranks lower in BENCH1-BENCH2.

Among the methods included only in BENCH1-BENCH2, CellCall, CytoTalk, Connectome, and Domino are ranked in the lower half in both benchmarks, while CellPhoneDBv2 holds around mid-table in both studies. ICELLNET ranks among the top methods in BENCH2 and in the top third of the ranking in BENCH1. NicheNet holds a good position in BENCH2 but ranks lower in BENCH1.

In terms of robustness, the two studies evaluating this aspect (BENCH1 and BENCH3) assign very different rankings to CellChat (most robust method in BENCH1, ranked lower in BENCH3) and CytoTalk (best tool in BENCH3, among the lowest in BENCH1). Both studies agree in placing NicheNet and scMLnet at the lower end of the ranking. Conversely, tools like NATMI and iTALK are consistently ranked as having among the best robustness in both studies, with NATMI reported as one of the top-performing tools.

Notably, two tools identified by BENCH1 as the most accurate, RNAMagnet and scSeqComm, were not evaluated in other studies. Similarly, BENCH3 highlighted Kumar and Skelly as highly robust, yet these were absent from the other three benchmarks. This underscores how top-performing tools may be missing in some benchmarking studies, and the best practice of prioritizing tools identified as top-performing in prior studies when evaluating all available tools is impractical.

This aggregated analysis of the tools, while informative, should be interpreted with caution (Supplementary Section S5). First, the ranking reflects relative performance rather than absolute efficacy. Second, evaluation methods vary across studies, employing different metrics or analyzing distinct aspects of the tools. Third, variations in tool versions, input parameters, and (mainly) LR databases used in different studies significantly influence performance.

## Future directions of cell–cell communication analysis

### C‌CC analysis on time series data

Current CCC approaches, where the time variable is considered, can be divided in two broad categories: developmental studies and longitudinal studies. In a seminal work, Schiebing et al. [94] proposed WaddingtonOT, a tool that explores optimal transport data in time dependent iPSC cells. By formulating the transport map between samples (individual time observations) in a Markov Chain fashion, WaddingtonOT can identify cell development related CCC events. Within the same category, TraSig uses a Continuous State Hidden Markov Model to identify cell development patterns (pseudotime) in scRNA-seq data, that are used to reveal pseudotime-dependent CCC events. In the context of longitudinal studies, MultiNicheNet, Tensor-cell2cell and scACCorDioN [95] are prominent tools that can compare sample level scRNA-seq datasets using CCC programs. Despite the availability of such tools, the analysis of CCC on time series data is still in its infancy, both in term of validation of the inferred processes and method development.

### Challenges and advances in modeling intracellular signaling networks in CCC

Modeling intracellular signaling networks is challenging due to their complexity and interconnected nature. This review highlights the need for improved data curation, incorporating high-resolution, high-confidence information on regulatory signals, including species-specific and tissue-specific pathway databases.

CCC involves intricate, recursive pathways linking intracellular processes to extracellular changes. As external factors or cellular contexts change, these signaling networks dynamically adapt cell responses. While simplifications in their modeling and annotation are often necessary, understanding the dynamic behavior of these pathways is crucial. For example, existing pathway databases lack comprehensive annotations of protein roles (e.g. repressor or activator), yet this information would be highly valuable for improving computational modeling of intracellular signaling. Improved pathway annotations should also include ions and metabolites, which interact with proteins to elicit responses.

An additional layer of complexity in CCC tools is the selection of signaling start and endpoints. Capturing the initiation of intercellular communication, such as ligand expression regulation, is crucial for linking upstream signaling events in one cell to downstream responses in another, as highlighted by CytoTalk. Moreover, most CCC tools focus on transcriptomic changes as only intracellular signaling endpoints, ignoring effects on other cell organelles (e.g. mitochondria, endoplasmic reticulum, lysosomes). More comprehensive approaches that integrate both upstream and downstream pathways would significantly enhance current CCC intracellular analysis.

### Multi-omics data integration in CCC

CCC involves interactions between diverse ligands and their specific receptors, varying in structure and chemical nature, including peptides, nucleic acids, lipids, and metabolites. Most CCC tools are limited to transcriptomics, neglecting non-peptide ligands and post-transcriptional modifications affecting protein activity. An exception is NeuronChat [10], which estimates neural communication based on non-peptide ligands using gene expression of synthesizing enzymes and vesicular transporters.

With the rapid development of new technologies, multi-omics data integration is emerging as a valuable approach for CCC analysis. Multimodal omics techniques simultaneously profile multiple types of molecules within a single cell, such as transcriptomics with proteomics [96,97] or chromatin accessibility [98], offering deeper biological insights despite lower cell-throughput than unimodal methods [99]. A prime example is given by the measurement of open chromatin accessibility at the single-cell level using single-cell ATAC sequencing (scATAC-seq) coupled with scRNA-seq data [100]. Tools like Cell Oracle [101], SCENIC+ [102], and scMega [103], infer regulatory networks using both scATAC-seq and scRNA-seq, enhancing transcription factor activity analysis and serving as data-driven foundations for many methods (e.g. decoupleR [104]). These approaches have the potential for advancing intracellular signaling studies in CCC analyses.

Mass spectrometry (MS)-based techniques have transformed proteome and metabolome profiling, enabling detailed analysis of protein composition, abundance, structure, function, and post-translational modifications [105]. In protein-based CCC, post-translational modifications like glycosylation affect LR affinity and binding and, consequently, signaling outcomes [1]. The integration of glycomic data, along with the use of dedicated databases of metabolite-protein interactions [106], represents a promising strategy to refine CCC predictions. Additionally, MS-based single-cell metabolomics has advanced, enhancing metabolite detection, sensitivity, annotation reliability, quantification accuracy, while also enabling spatial mapping of metabolites within their tissue of origin [99]. While extracellular MS holds clear value for investigating ligands, low concentrations of target metabolites and proteins challenge sample preparation. However, technological advancements and improved enrichment methods are expected to expand extracellular MS-based omics, complementing transcriptomics in CCC research.

### De novo discovery of new interactions

Most CCC tools rely on current LR databases for inferring ongoing CCC, however this approach presents multiple challenges. First, databases may be incomplete, missing newly discovered or context-specific LR pairs. Additionally, databases are often built on curated information from limited studies, introducing biases and failing to account for variations across tissue types, developmental stages, or species. Ligand-receptor interactions are highly dynamic and can vary based on environmental factors, and cellular states, none of which are easily captured and annotated in current databases. Finally, extensive experimental validation of predicted interactions remains resource-intensive and complex, limiting comprehensive assessment of existing databases. These limitations underscore the need for advanced approaches that go beyond existing databases and instead infer CCC networks directly from high-throughput experimental data. Emerging database-agnostic methods enable the inference of CCC directly from gene expression data, making interaction discovery possible across all organisms without relying on predefined LR databases. Further, recent structure-based CCC approaches have made it possible to predict potential novel LR pairs using protein structure data (e.g. CellDialog). These methods offer a promising link to cutting-edge generative models like ESM3, holding significant potential for the identification and engineering of novel LR interactions. Despite progress, major unresolved challenges remain, including the combinatorial complexity of interactions, the absence of a centralized data-sharing infrastructure, and insufficient communication between research groups.

## C‌CC-catalog

To gather and share comprehensive, up-to-date knowledge on CCC, we created CCC-Catalog (https://sysbiobig.gitlab.io/ccc-catalog), an online resource compiling a vast array of available CCC tools and resources.

CCC-Catalog serves as a centralized hub for CCC analysis utilizing scRNA-seq and ST data, and it invites users to actively contribute by keeping the catalog updated and continually enhancing its resources.

The CCC resources page includes all major LR databases and more for Human, with some resources available for other species (e.g. Mouse, Zebrafish). Using detailed filters, users can search resources by type and number of annotated interactions (intercellular or intracellular), availability of protein complex data, and additional attributes.

The CCC tools page features a thorough catalog of CCC methods, allowing users to refine searches based on input data type (e.g. scRNA-seq, spot-based ST), preferred programming language and software format (command-line tool, R package, web app), types of CCC signaling analyzed (intercellular, intracellular, or both), visualization options (e.g. circos plots, heatmaps), and advanced features (e.g. differential CCC analysis, time series analysis).

The literature page provides summaries of the latest studies on CCC tools and methodologies, including reviews, comparative studies, and benchmarking analyses.

## Conclusions

The field of CCC analysis has advanced significantly, providing researchers with a vast array of bioinformatics tools and resources. A thorough understanding of these tools and resources, including their strengths, limitations, and underlying assumptions, is essential for conducting reliable and robust bioinformatics analyses. This study aims to provide a comprehensive review of the current state of the art in CCC analysis from scRNA-seq and ST data, highlighting the heterogeneity of existing methods and databases while providing a detailed characterization of their features.

Furthermore, resources like CCC-Catalog serve as invaluable platforms for sharing tools, databases, and literature, fostering accessibility and collaboration within the research community. By embracing these advancements and addressing current limitations, the field can develop a deeper, more nuanced understanding of CCC, and uncover new insights into fundamental biological processes and their implications for health and disease.

Despite considerable progress in CCC analysis, our findings confirm that many challenges remain. The lack of ground truth and the difficulty in systematically evaluating CCC inference tools are the major challenges. Addressing this gap is essential for advancing the field toward more comprehensive and reliable analytical frameworks. Additionally, as transcriptomics dataset continue to grow and experimental design become more complex, future CCC tools must prioritize computational efficiency and effective visualizations to handle large-scale data more seamlessly.

In terms of prior knowledge, improving existing LR databases and developing computational methods to predict unannotated LR interactions would significantly advance the field. At the same time, recent database-agnostic approaches are examples that open new venues for de novo discovery of CCC interactions. Additional benchmarks and methods are still required in this regard. For intracellular analysis, improved data curation in signaling pathways, incorporating species-specific and tissue-specific pathway databases, refining protein functions annotation, and integrating upstream and downstream pathways, is necessary for more robust CCC analysis.

The incorporation of multi-omics data, including transcriptomics, proteomics, glycomics, and metabolomics, presents another promising avenue for enhancing CCC inference. Technologies like mass spectrometry and single-cell multi-omics offer unprecedented opportunities to capture the diverse molecular layers of cell signaling, improving both accuracy and comprehensiveness of CCC analyses.

**Key Points**

- The field of cell–cell communication (CCC) analysis has advanced significantly, offering researchers a vast array of bioinformatics tools and resources, while also posing the challenge of selecting the most suitable tool/resource for their specific needs.
- We comprehensively characterize 58 CCC bioinformatics tools and 26 databases, providing a detailed assessment of their strengths, limitations, and underlying assumptions, all of which are crucial for conducting reliable and robust bioinformatics analyses.
- We reviewed nine prior reviews, two comparative studies, and four benchmarking studies, identifying consistent trends across diverse tools and databases. By aggregating insights from existing literature, we highlight overarching patterns and emerging directions in CCC analysis.
- We discuss key challenges affecting state-of-the-art tools, including validation difficulties, computational efficiency concerns, and challenges in interpreting and visualizing results, and explore emerging trends, such as time-series analyses, multi-omics integration, and machine learning-based predictions of novel interactions.
- To streamline access to these extensive resources, we developed CCC-Catalog (https://sysbiobig.gitlab.io/ccc-catalog), an interactive online platform that enables researchers to filter and visualize tools and databases based on their specific needs, simplifying the process of identifying the most appropriate resources for CCC analysis.

## Supplementary Material

Supplementary_Data_1_bbaf280

Supplementary_Data_2_bbaf280

Supplementary_materials_bbaf280

## Data Availability

The data underlying this article are available in the article and in its online supplementary material.
