# Supplementary material for: Advances and challenges in cell–cell communication inference: a comprehensive review of tools, resources, and future directions
Source: Brief Bioinform. 2025 Jun 19;26(3):bbaf280. doi: 10.1093/bib/bbaf280 (PMC12204611; doi:10.1093/bib/bbaf280)
Supplement: Supplementary_materials_bbaf280 [file supplementary_materials_bbaf280.docx]

**Advances and Challenges in Cell-Cell Communication Inference: A Comprehensive Review of Tools, Resources, and Future Directions**

Giulia Cesaro ^ (1), James Shiniti Nagai ^ (2), Nicolò Gnoato ^ (3), Alice Chiodi ^ (4), Gaia Tussardi ^ (1), Vanessa Klöker ^ (2), Carmelo Musumarra (3), Ettore Mosca (4), Ivan G Costa (2), Barbara Di Camillo (1), Enrica Calura ° (3), Giacomo Baruzzo ° * (1)

^ Authors contributed equally

° Authors contributed equally

* Corresponding author

1. Department of Information Engineering, University of Padova, Padova, Italy
2. Institute for Computational Genomics, RWTH Aachen Medical Faculty, Aachen, Germany
3. Department of Biology, University of Padova, Padova, Italy.
4. Institute of Biomedical Technologies, National Research Council (CNR), Segrate (Milan), Italy

#

*Supplementary Materials*

**Supplementary Sections 2**

Section S1 - Ligand-receptor databases 2

Section S2 - CCC tools 3

Section S3 - CCC tools taxonomy 4

Section S4 - CCC literature 5

Review studies 5

Comparison studies 7

Benchmarking studies 8

Section S5 - Aggregated analysis of current benchmarking studies 11

Xie et al. (Biomolecules, 2023) 11

Zhang et al. (Frontiers in Genetics, 2023) - RobustCCC 11

Luo et al. (Genome Research, 2023) - ESICCC 12

Liu et al. (Genome Biology, 2022) 13

Interpretation of the aggregated analysis of the four benchmarking studies 13

**Supplementary Figures 15**

**Supplementary Tables 17**

**Supplementary References 32**

##

# **Supplementary Sections**

## **Section S1 - Ligand-receptor databases**

To compile a comprehensive set of LR databases relevant to CCC (Supplementary Data 1), we adopted a systematic approach to ensure the integration of accurate and widely utilized resources. First, we conducted an extensive review of primary research articles describing newly developed tools for CCC analysis. These articles often provided detailed information about the ligand-receptor databases implemented or referenced in their methodologies, offering a valuable starting point for our selection process. Additionally, we reviewed existing literature, specifically focusing on comprehensive reviews dedicated to CCC analysis. This allowed us to cross-verify the databases cited in these reviews with those included in our work, ensuring that the resources we considered were both current and widely recognized in the field. Finally, we performed a targeted literature search using specific keywords such as *cell-cell communication* and *ligand-receptor databases*. This strategy was designed to identify any additional resources or studies that might not have been covered in the initial stages of our investigation. Through this multi-faceted approach, we were able to curate a robust collection of ligand-receptor databases that form the basis for the analyses presented in this study.

The curation of this collection was performed to obtain a list of databases that can be generally used, novel and accessible. Generality was achieved by maintaining only the databases with at least human interactions data, which can be mapped to any organisms through orthology. We thus discarded species-specific databases, like Kumar2018 [1], Baccin2020 [2] are all collection of mouse data, FlyPhoneDB [3] is developed on *Drosophila*, PlantPhoneDB [4] on plants, cell2cell-DB [5] on *Caenorhabditis elegans*, and Raredon2019 [6] on mammal data. We also excluded very specific collection, like Zheng2022 [7], that contains metabolite data, which are not used by the tools present in this review, and Kiroauc2010 [8] and NeuronChatDB [9], that contain data of very restrict case studies (blood stem cells the first, neuron communication the second), but that is worth mentioning.

Novelty was assured by searching the collection to maintain only the ones that use new knowledge, new manual curation of literature data, or a combination of the two. In case of the usage of literature data, we discarded every collection that represents: a filtering of already published resources based on case-specific data (Ding2016 [10], Cain2020 [11], Raredon2019 [6], EMBRACE-DB [12]); mapping through orthology but without manual curation (Yuzwa2016 [13], Skelly2018 [14]); merging of two or more databases, but again without any manual curation (Pavilcev2017 [15]). Considering accessibility, we maintained only collections available on-line at the present date (2024), as some are published but no more available (Choi2015 [16]). We also collected information about possibility to download (available on-line or only as built in a package) and usage (saved in widely used file formats or specific programming language files), but do not discriminate on these. The database table was completed by using the data (sources used, number of interactions, species, location of the database) of the latest version available (July 2024), to match the current state-of-the-art.

##

## **Section S2 - CCC tools**

To compile a comprehensive list of CCC tools relevant to single-cell and spatial data analysis, we first conducted an extensive literature review of primary research articles detailing newly developed tools specifically designed for these types of data. We then reviewed recent comprehensive reviews on CCC tools, cross-referencing the tools cited in these reviews with those already identified in our work. This review presents a curated list of tools for cell-cell communication analysis designed specifically for single-cell and spatial data analysis up until July 2024 (Supplementary Data 2). However, some tools, despite being included in recent reviews, were excluded from our list due to specific limitations or focus areas.

Notably, tools that are not designed for single-cell RNA sequencing or spatial transcriptomics data were excluded, as they focus on other data types. For instance, some tools are tailored for bulk RNA sequencing, such as CCCExplorer [17], LIRICS [18], bulkSignalR [19], and RaCinG [20]; others specialize in RNAmerscope data, such as STRISH [21]; or rely on mass spectrometry-based approaches, such as SEARCHIN [22].

Tools such as InterCellar [23] and TALKIEN [24] were excluded, as they are primarily web applications and do not provide novel methodological frameworks for CCC analysis. Additionally, Differential MultiNicheNet [25] was excluded because it is a modification of the existing MultiNicheNet tool [26], rather than a new, standalone method. LRLoop [27] was excluded as it addresses a niche area, focusing exclusively on identifying paired ligand-receptor interactions.

Other tools were excluded due to their focus on specific biological systems or mechanisms, such as NeuronChat [28], which targets neuronal networks, and MEBOCOST [29], which focuses on metabolite-mediated interactions. Some tools are species-specific, including FlyPhoneDB [3] and PlantPhoneDB [4]. Additionally, certain tools are tailored to work exclusively with particular datasets or lack general-purpose applicability, such as GCNG [30], MESSI [31], CellGiQ [32], SPRUCE [33], CCInx [34], SpaCET [35], and Clarify [36]. Some tools do not primarily focus on inferring ligand-receptor communication, such as Misty [37], Niches [38], NCEM [39], CCPLS [40], DIALOGUE [41], MOFAcell [42], scMultiSim [43], scITD [44], FunRes [45], CellNeighborEX [46], CSOmap [47], DeepLinc [48], Neighbor-seq [49], SPROUT [50], and STcomm [51].

Finally, tools were also excluded if their code is unavailable, undocumented, or non-functional. Examples include CellComNet [52], CellEnBoost [53], CellDialog [54], CINS [55], BATCOM [56], Topic Modeling [57], Calligraphy [58], HiVAE [59], and LR Hunting [60].

## **Section S3 - CCC tools taxonomy**

Aiming to provide an unified classification of the methodologies employed on the large amount of tools found in the current literature, here we propose the following taxonomy:

- **Network-based:** Network science derived methods/algorithms (e.g. Page Rank, Shortest Path) are used to infer intercellular and/or intracellular signaling.
- **Statistics-based:** Statistical tests (e.g. Differential expression, t-test, Fisher test, GSEA) are used to infer intercellular and/or intracellular signaling.
- **Correlation-based:** Correlation of gene expression values, or score derived from them, is used to infer intercellular and/or intracellular signaling.
- **Coexpression-based**: Coexpression patterns of genes expression values are used to infer intercellular and/or intracellular signaling.
- **Expression-based:** Threshold of the gene expression values or arithmetic operations on gene expression values are used to determine intercellular and/or intracellular signaling.
- **Machine Learning-based:** Classification or regression models are trained to predict intercellular and/or intracellular signaling.
- **Space-based:** Spatial coordinates are used to infer intercellular and/or intracellular signaling.
- **Matrix decomposition-based**: Matrix/Tensor decomposition models are used to infer the intercellular signaling.

We assigned to each tool the taxonomy according to the method description. Note that one tool can be assigned to multiple taxonomies, i.e. it can use more than one class of methodologies to model/infer CCC. Moreover, we observed an increase of tools with multiple taxonomies in recent years. This corroborates with the sophistication of the advances in the CCC field not only in the data acquisition (sequencing and imaging technologies) but also in the development of inference methods.

##

## **Section S4 - CCC literature**

### Review studies

**Shao et al. (2020) “New avenues for systematically inferring cell-cell communication: through single-cell transcriptomics data”, Proteins & Cell**

This review examines 16 CCC tools, categorizing them by two main strategies: ligand-receptor interaction for chemical signaling and physical contact-based communication through structures like gap junctions. Although no ligand-receptor databases are mentioned, the review underscores CCC's relevance across a wide array of biological contexts, including liver diseases, inflammation, and drug resistance. It highlights both the strengths and limitations of scRNA-seq in CCC inference, especially the mismatch between mRNA and protein expression. Spatial transcriptomics and advanced computational models are identified as crucial next steps for more precise CCC analysis.

**Ma et al. (2021) “Applications and analytical tools of cell communication based on ligand-receptor interactions at single cell level”, Cell & Bioscience**

Ma et al. offer an overview of 14 tools and 25 databases, categorizing tools into general analysis (exploring ligand-receptor interactions without prior knowledge of cell types), signal pathway analysis (which combines ligand-receptor data with pathway analysis), and spatial cellular communication (which reconstructs spatial information from ligand-receptor interactions). The review emphasizes the importance of advanced analytical techniques such as expression levels, tensor decomposition, and cell-cell similarity matrices, and discusses their applications in healthcare, especially in cancer research, pathogenic infections, and pharmacological studies.

**Almet et al. (2021) “The landscape of cell-cell communication through single-cell transcriptomics”, Current Opinion in Systems Biology**

Almet et al. discusses 19 CCC tools, categorizing them by their input data requirements (scRNA-seq or ST data) and their consideration of downstream responses and multi-subunit complexes. While specific ligand-receptor databases are not mentioned, the review indicates that these tools rely on curated databases of ligand-receptor pairs. Several visualization methods, such as circle plots, chord diagrams, and bubble plots, are highlighted to represent cell signaling patterns. The tools are applied in diverse fields, including hair follicle development, brain-barrier signaling during COVID-19, immune regulation in pregnancy, and wound healing. The review also addresses the limitation of scRNA-seq-based tools, which can produce false positives due to lack of spatial context in CCC inference.

**Armingol et al. (2021) “Deciphering cell-cell interactions and communication from gene expression”, Nature Reviews Genetics**

Armingol et al. cover 17 CCC tools, categorizing them based on computational strategies: differential combination-based, network-based, expression permutation-based, and tensor-based methods. They emphasize the critical role of well-curated ligand-receptor databases, providing a list of 32 databases that support CCC analysis. The review also discusses various methodologies employed by these tools to mitigate false positives and negatives, such as permutation-based analyses, subsampling and enrichment techniques. Additionally, it highlights the potential for integrating multi-omics data, particularly proteomics and glycomics, to enhance predictions of ligand-receptor interactions. The authors stress the importance of considering spatial and temporal factors in CCC analysis, noting that traditional transcriptomics-based methods may not capture these dynamics accurately. Overall, the review provides a comprehensive overview of the current landscape of CCC tools and their applications in understanding intercellular communication.

**Bridges et al. (2022) “Mapping and Validation of scRNA-Seq-Derived Cell-Cell Communication Networks in the Tumor Microenvironment”, Frontiers in Immunology**

Bridges et al. provide a comprehensive review of the application and validation of CCC tools within the TME, focusing on how insights derived from scRNA-seq can elucidate immunosuppressive and pro-inflammatory signaling mechanisms in cancer. The review emphasizes the importance of validating predicted signaling networks through functional experiments rather than merely comparing or benchmarking CCC tools. The authors analyze 14 key tools utilized in cancer research, categorizing them based on their approaches to CCC inference, which include upregulation matching, statistical frameworks, and network-based models. They underscore the significance of well-curated ligand-receptor databases and highlight the variations in their scope and coverage. Moreover, the review discusses experimental validation techniques, particularly within the field of immuno-oncology, detailing methods such as protein secretion assays and immunostaining to verify predicted signaling pathways against biological data. While the authors do not offer a formal classification of the tools or detailed scoring models, they highlight the challenges of validating CCC in complex, immunosuppressive environments and advocate for more extensive validation frameworks. Additionally, visualization techniques are mentioned, with a particular emphasis on the user-friendly outputs from tools, aimed at linking CCC insights to immunosuppressive and pro-inflammatory interactions in cancer research.

**Jin et al. (2022) “Computational exploration of cellular communication in skin from emerging single-cell and spatial transcriptomic data”, Biochemical Society Transaction**

Jin et al. review the role of CCC inference in tissue development, homeostasis, disease, and aging, with a particular focus on its applications and potential in skin-related data. Rather than conducting a systematic review of CCC tools and databases, the authors cite 28 tools, covering both scRNA-seq and spatial transcriptomics applications, and describe the functionality of several prominent examples.

**Peng et al. (2022) “Cell–cell communication inference and analysis in the tumour microenvironments from single-cell transcriptomics: data resources and computational strategies”, Briefings in Bioinformatics**

Peng et al. conducted an in-depth review of CCC tools and resources for inferring interactions within tumor microenvironments, covering 11 CCC resources and 18 CCC tools. For each database, the authors assessed the number of annotated ligands, receptors, and ligand-receptor pairs, as well as the primary data sources used to build the database. In evaluating CCC tools, they documented required input data and the scoring schemes adopted. Each tool was classified into one of four categories: network-based, machine learning-based, spatial-based, or other, with advantages and limitations of each approach highlighted. Peng et al. also examined CCC data visualization tools and the validation strategies used by the tool developers, categorizing them into experimental methods (e.g., proteomics, microscopy with immunostaining), computational approaches (e.g., statistical testing), and alignment with established biological literature.

**Wang et al. (2023) “The promising application of cell-cell interaction analysis in cancer from single-cell and spatial transcriptomics”, Seminars in Cancer Biology**

Wang et al. review 25 CCC tools for cancer data analysis. For each tool, the authors document whether it operates at the intercellular or intracellular level, the type of output score (continuous or binary), and the methodologies used for intercellular (threshold-based, differential expression-based, permutation-based, coexpression-based, optimal transport, neural network) and intracellular analyses (Fisher’s exact test, differential expression-based, network-based, coexpression-based). Additionally, they note the visualization type and programming language for each tool.

**Armingol et al. (2024) “The diversification of methods for studying cell-cell interactions and communication”, Nature Review Genetics**

Armingol et al. provides a comprehensive review of CCC tools, encompassing both general-purpose and context- or organism-specific tools, as well as CCC data visualization applications and tools that incorporate CCC analysis for additional bioinformatics tasks (e.g., cell colocalization analysis, data simulation). Their review covers an impressive 104 tools related to CCC. The authors classify these tools by their underlying methodology (rule-based, data-driven, or hybrid) and their primary functionality, categorizing them as follows: 'core tools' (general frameworks), 'finer' (single-cell level CCC), 'deeper' (including intracellular analysis), 'broader' (accommodating multiple conditions), 'more localized' (integrating spatial information), and 'other improvements' (e.g., score aggregation, simulation-based methods, interactive interfaces).

### Comparison studies

**Dimitrov et al. (2022) “Comparison of methods and resources for cell-cell communication inference from single-cell RNA-Seq data”, Nature Communications**

LIANA established a platform for the comparative analysis of multiple cellular communication tools, offering a resource to manage the heterogeneity of input data and outputs across different tools, facilitating a more structured comparison. The study systematically compared 7 CCC methods and 16 CCC resources.

In terms of CCC resources, there is significant variation in the number of included ligands, receptors, and ligand-receptor pairs, compared to the uniqueness of the included molecules. The authors noted limited uniqueness across resources (an average of ~10% unique ligand-receptor pairs), with Cellinker being the exception (~39% unique ligand-receptor pairs). Among the reviewed CCC resources, a high similarity in included ligand-receptor pairs was observed across CellTalkDB, ConnectomeDB, iTALK, LRdb, and Ramilowski. Interestingly, the LIANA study found higher similarity across resources when considering ligands and receptors rather than ligand-receptor pairs. This suggests that different CCC resources often include the same molecules but report different interactions between them. Remarkably, the authors identified that different CCC resources are biased towards certain pathways and functional domains. This makes the choice of CCC resources a major source of bias in the functional interpretation of CCC results.

In terms of CCC tools, the LIANA study revealed a low overlap among the predicted ligand-receptor interactions, even when considering only the top-rated interactions. This discrepancy is mainly due to the different nature of the tools, each capturing distinct aspects of CCC to score and prioritize the inferred interactions.

The authors reported that the methods are generally robust to low number of cells per cluster, whereas erroneous cell type annotations had a more pronounced effect on the results. Introducing false ligand-receptor pairs in the input resources showed a minimal impact on method performance, with the methods still able to preserve the highest-ranked interactions. Overall, the authors identified CellChat, CellPhoneDB and SingleCellSignalR as the more robust tools against noise in transcriptomics data and input CCC resources, despite all tools generally showing stable performance.

**Wang et al. (2022) “A systematic evaluation of the computational tools for ligand-receptor-based cell–cell interaction inference”, Briefings in Functional Genomics**

In the study by Wang et al., nine tools (iTALK, ICELLNET, NicheNet, NATMI, SingleCellSignalR, CellChat, PyMINEr, CellPhoneDB, and scMLnet) were compared for LR interaction inference and the construction of CCI networks using well-characterized scRNA-seq datasets. The aim was to analyze differences in LR resources, prediction accuracy, robustness to subsampling and clustering resolution, memory consumption, and runtime. The LR database each tool relies on is a major factor influencing CCI predictions. While iTALK, ICELLNET, and NATMI showed higher consistency in the number of predicted interactions, the overlap between specific LR pairs was relatively low (e.g., iTALK shared only 12.8% of its predictions with ICELLNET), suggesting significant variability in prediction algorithms and filtering strategies, potentially introducing false positives. On the other hand, CellChat and SingleCellSignalR produced fewer LR pairs, which may reduce noise but risk missing biologically relevant interactions. To assess predictive accuracy, the predicted LR interactions were benchmarked against C-LRI, a curated database of well-annotated LR pairs. iTALK, scMLnet, and CellChat demonstrated strong matches with C-LRI, while tools like NicheNet, CellPhoneDB, and PyMINEr had lower overlap, raising concerns about their prediction reliability. NATMI’s predictions were most consistent with C-LRI, indicating its higher likelihood of capturing well-annotated LR interactions. In terms of robustness, tools like ICELLNET, iTALK, and NATMI maintained relatively stable predictions even when input data were subsampled or cell clustering resolution was adjusted, whereas scMLnet and SingleCellSignalR showed greater sensitivity to data reduction, leading to fewer predicted interactions. Computational efficiency also varied widely across tools. iTALK was the fastest, completing analyses within 20 seconds, while scMLnet took up to 8 hours within the same dataset, reflecting the complexity of its network construction and visualization features. Memory consumption ranged from 0.5 to 2.5 GB for most tools, though PyMINEr (13.3 GB) and SingleCellSignalR (5.2 GB) were exceptions, potentially limiting their use in large-scale studies.

Overall, this comparison highlights the heterogeneity of LR-based CCI prediction tools in terms of both prediction accuracy and computational efficiency. Tools like iTALK, ICELLNET, and NATMI generate a high volume of predictions, while CellChat and SingleCellSignalR adopt a more conservative and potentially more robust approach. Employing multiple tools in tandem could be a valuable strategy for obtaining more accurate CCI predictions.

### Benchmarking studies

**Luo et al. (2023) “ESICCC as a systematic computational framework for evaluation, selection, and integration of cell-cell communication inference methods“, Genome Research**

The ESICCC framework, introduced by Jiaxin Luo et al., provides a systematic approach to evaluate, select, and integrate CCC inference methods, benchmarking 18 LR inference tools and 5 LR-target tools across 116 datasets. It is the most extensive and comprehensive study in terms of the number of tools analyzed, tested aspects (accuracy, robustness and usability), and scenarios considered. The authors identify CellChat, ICELLNET, NATMI, RNAMagnet, and scSeqComm as the five tools offering the best overall balance between accuracy, robustness, and usability. In particular, CellChat, RNAMagnet, and scSeqComm excel in prediction accuracy, with CellChat also excelling in result stability alongside NATMI, while RNAMagnet demonstrates excellent usability. The study emphasizes the importance of computational costs, as tools like CellChat demonstrated high accuracy but consumed more memory and runtime, while NATMI and CellTalker delivered robust predictions with lower resource requirements. The study highlights that some methods showed performance fluctuations depending on the dataset characteristics, underscoring the need for careful method selection based on specific research requirements.

**Zhang et al. (2023) “RobustCCC: a robustness evaluation tool for cell-cell communication methods”, Frontiers in Genetics**

Zhang et al. work evaluated 14 tools based on the robustness of their results to various sources of variability and noise, such as data variability (i.e., batch effects and biological replicates), transcriptomic noise (i.e., noise in expression levels and dropout events), and noise in prior knowledge (i.e., errors in cell cluster annotations and ligand-receptor databases). The authors assess the robustness of the methods both against individual sources of variability and noise, as well as providing an overall aggregated robustness metric. They identify CytoTalk, Kumar, NATMI, iTALK, and Skelly, in that order, as the top methods. The study highlights the importance of selecting tools based on dataset characteristics, with findings of low consensus among tools and varying computational demands, noting that CytoTalk’s robustness comes with high resource requirements, making careful tool selection essential. Another important aspect of the study is its examination of the overall agreement between different CCC methods. The results indicate a low level of consensus, with the mean pairwise Jaccard index ranging from 0.003 to 0.183 across simulated datasets. This observation is consistent with findings from the LIANA study, which reported similarly low agreement, reinforcing the idea that different methods may produce varying results depending on the dataset and conditions. The study underscores the need for careful method selection based on the specific characteristics and requirements of the data being analyzed, as no single method is universally superior across all scenarios.

**Liu et al. (2022) “Evaluation of cell-cell interaction methods by integrating single-cell RNA sequencing data with spatial information” Genome Biology**

Liu et al. study analyzed 15 tools in terms of concordance of predicted CCI and accuracy, using both 5 real and 15 simulated dataset. Authors classified CCIs into long-range and short-range interactions based on the spatial distribution of ligand-receptor pairs observed in ST datasets, and focused on measuring the coherence between expected and observed spatial interaction distances, as well as the consistency of predicted CCIs across different methodologies. The authors identified CellChat, ICELLNET, NicheNet, CellPhoneDBV2, and SingleCellSignalR as the top tools in terms of accuracy. The study also assessed runtime and memory, identifying SingleCellSignalR as effective but resource-intensive, while CellChat and CellPhoneDB offer high-confidence results and practical resource management. Additionally, further evaluation showed that the statistical-based tools generally outperformed both network-based and ST-based methods, consistently achieving higher F1 scores in overlap analysis with simulated CCIs, highlighting their reliability in predicting interactions accurately. The study reveals that CCI predictions vary significantly across tools, with statistical-based methods showing greater agreement with one another and superior efficiency in both time and memory consumption. The authors recommend combining the results of multiple CCI tools to enhance the accuracy and reliability of identified interactions.

**Xie et al. (2023) “A Comparison of Cell-Cell Interaction Prediction Tools Based on scRNA-seq Data”, Biomolecules**

Xie et al. analyzes 7 CCC tools and a consensus score provided by LIANA framework in terms of prediction accuracy. The benchmark is built around a gold standard dataset of idiopathic pulmonary fibrosis related CCIs, curated from the literature, to provide a reliable framework for comparison. The results indicate that all tools perform adequately in predicting source-target, i.e. cell-cluster pair, interactions, but struggle with ligand-receptor resolution predictions, demonstrating the challenge of achieving accurate predictions with current methodologies. The authors recommend CellPhoneDB and NATMI based on F1-score and MCC. They also highlight CellPhoneDB and scMLnet as the tools with high precision and specificity, while NATMI and CellPhoneDB achieve high sensitivity. Despite the strong overall performance of CellPhoneDB, no single tool emerged as a universal solution, with each exhibiting unique strengths depending on the study's focus and constraints.

**Initiative “Open Problems in Single cell“**

The open problems initiative (<https://openproblems.bio>)[61] attempts to provide a structured resource for researchers for comparing computational tools dedicated to several bioinformatics analysis. It offers standardized datasets and encourages researchers to contribute their methods and insights, fostering a collaborative approach to addressing open problems in the field. Among the ongoing benchmarking initiative, there are two dedicated to cell-cell communication analysis:

- Cell-Cell Communication Inference (Ligand-Receptor level) [<https://openproblems.bio/results/cell_cell_communication_ligand_target/>]: This benchmarking evaluates the methods’ ability to predict cytokines related interactions, using a set of cytokine activities, inferred using a multivariate linear model with cytokine-focused signatures, as assumed true cell-cell communication.
- Cell-Cell Communication Inference (Cell cluster level) [<https://openproblems.bio/results/cell_cell_communication_source_target/>]: This benchmarking evaluates tools in their ability to predict interactions between spatially-adjacent cell clusters, using adjacent cell types as assumed benchmark truth.

Both initiatives are assessing several CCC tools (CellPhoneDB, SingleCellSignalR, Connectome, NATMI) and custom scoring schemes, evaluating the accuracy in terms of Precision-Recall AUC and the computational burden in terms of execution time and memory usage.

##

## **Section S5 - Aggregated analysis of current benchmarking studies**

This section contains a detailed summary of the four CCC tools benchmarking studies analyzed in the main manuscript (Section 4.3). For each study, this section reports the tested tools and resources, describes the overall assessment framework and reported ranking, and identifies the tools suggested by the authors. Such information was used to create Figure 3 in the main manuscript and to perform the aggregated analysis of the four benchmarking studies.

### Xie et al. (Biomolecules, 2023)

Tested tools: CellChat (v.1.1.3), iTALK (v.0.1.0), SingleCellSignalR (v.1.8.0), CCInx (v.0.5.1), scMLnet (v.0.1.0), CellPhoneDB (v.2.0.0), NATMI and LIANA-Ensemble (v.0.1.12 - Ensemble score based Connectome, NATMI, CellPhoneDB, SingleCellSignalR, and iTALK).

Tested LR resource: LIANA ensemble database (CellPhoneDB, CellChatDB, ICELLNET, connectomeDB2020, and CellTalkDB)

Assessment: Authors generate a list of source-target and ligand-receptor interactions for one specific disease (idiopathic pulmonary fibrosis). They evaluate the CCC tools in the ability to identify such interactions in 4 scRNA-seq datasets of idiopathic pulmonary fibrosis (GSE122960, GSE128033, GSE135893, and GSE136831), computing Precision, Sensitivity, Specificity, F1-score, and MCC. Authors evaluate the performance of the methods in 2 models:

- ST (source-target) model counts the number of LR interactions in each cell pair, so ignoring from which ligand-receptor pairs it comes from

- STLR (source-target-ligand-receptor) model maintains the information on the source, target, ligand, and receptor for each CCI, so assessing the single interactions.

Reported ranking: Authors do not provide an overall rank of the tools in their work. We ranked the tools based on the combined evidence of the reported F1-Score and MCC (Figure 3 of the original manuscript) for the STLR model. As done by the authors in the discussion of the results, we choose F1-Score and MCC to describe the overall performance of the methods, since they provide a more complete view of global performance compared to Precision, Sensitivity, or Specificity. We choose the STLR model since it is the most informative one, and the more closed to CCC output.

Suggested tools: The authors recommended CellPhoneDB and NATMI based on F1-score and MCC. They also identify CellPhoneDB and scMLnet as the tools with high precision and specificity, while high sensitivity is achieved by NATMI and CellPhoneDB. These recommendations are reported in section 4.1 Tool Recommendation in the original manuscript.

### Zhang et al. (Frontiers in Genetics, 2023) - RobustCCC

Tested tools: CellPhoneDB (v4.0.0), CellCall (v1.0.7), CellChat (v1.1.3), ICELLNET (v1.0.1), iTALK (v0.1.0), Kumar, NATMI (commit f35f677), scConnect (v1.0.3), Skelly, SingleCellSignalR (v1.6.0), Zhou, CytoTalk (v4.0.10), NicheNet (v1.0.0), scMLnet (v0.1.0).

Tested LR resource: LIANA ensemble database (CellPhoneDB, CellChatDB, ICELLNET, connectomeDB2020, and CellTalkDB)

Assessment: Authors collect single-cell RNA-seq data of mouse brains from the Single Cell Portal (ID: SCP795). Such scRNA-seq data were used by authors to simulate 6 main scenarios: two types of replicated data (biological replicates and simulated replicates), two types of transcriptomic data noise (Gaussian noise and dropout), and two types of noise in prior knowledge (cell type permutation and ligand-receptor permutation). They evaluate the tools in the robustness of predicted CCI across the simulated dataset, at varying level of noise (e.g. increasing amount of dropouts), computing the Jaccard Index between the CCI predicted on the original dataset and the CCI predicted on the simulated dataset at varying level of noise.

Reported ranking: Authors provide the ranking of the tools in Figure 3 and in Supplementary Table 7. The ranking is computed based on the overall average Jaccard Index across all the simulated datasets.

Suggested tools: The authors identified in CytoTalk the most robust method, followed by Kumar. Overall, the authors identified CytoTalk, Kumar, NATMI, iTALK and Skelly as the most robust tools. These indications are available in section “3.4 Ranking CCC methods based on robustness” in the original manuscript.

### Luo et al. (Genome Research, 2023) - ESICCC

Tested tools: CellPhoneDB v2.0, CellPhoneDB v3.0, CellTalker, Connectome, ICELLNET, NATMI, iTALK, scConnect, SingleCellSignalR, RNAMagnet, CellChat, scSeqComm, NicheNet, CytoTalk, Domino, CellCall, scMLnet, PyMINEr.

NOTE: Authors tested also CCC tools that can infer ligand-receptor-targer interactions, namely NicheNet, CytoTalk, scMLnet, stMLnet, MISTy, and HoloNet. However, they performed a limited number of tests on this former class of methods and did not include them in the final tool ranking. For this reason, we exclude them from our analysis.

Tested LR resource: Each tool was run with a different LR database. Details are available in supplementary section S4 of the original manuscript.

Assessment: Authors evaluated the 18 CCC tools based on 3 main criteria: accuracy, robustness and usability.

Accuracy:

- DLRC (differential LR correlations) in ST data: authors computer CCI interactions on scRNA-seq datasets, and then computed CCI on matched ST datasets by evaluating the difference of correlations of LR pairs (DLRC) in the group of close cell pairs and distant cell pairs.

- agreement with CAGE data: authors defined LR interaction with the expressions of ligand and receptor in the respective cell types both >10 TPM as active CCI, and evaluate the performance of the CCC tools in identifying such interactions using AUPRC and AUROC.

- agreement with proteomics data: authors defined LR interaction with the average counts of unique peptides of ligand and receptor greater than 2 as active CCI, and evaluate the performance of the CCC tools in identifying such interactions using AUPRC and AUROC.

Robustness: authors subsampled 14 scRNA-seq datasets at different sampling rates, and compared the predicted CCI on subsampled dataset with the ones on original datasets as a function of Jaccard Index.

Usability: authors define a custom scale to measure execution time and RAM usage in range [0-1]

Reported ranking:

Authors provide the ranking of the tools in terms of each evaluated aspect:

- Accuracy: DLRC ranking, CAGE ranking, proteomics ranking, overall accuracy ranking (i.e. average ranking computed from the previous 3 ranking)

- Robustness

- Usability: memory usage ranking, CPU time ranking, overall usability ranking (i.e. average ranking computed from the memory usage ranking and CPU time ranking)

Moreover, the authors computed the overall ranking as the average of the overall accuracy ranking, the robustness ranking and the overall usability ranking.

In our analysis, we reported the overall accuracy ranking, robustness ranking, the overall usability ranking and the overall ranking.

Suggested tools: The authors identified RNAMagnet, CellChat, and scSeqComm as the best methods in terms of overall accuracy. Considering also stability and usability, the authors identified CellChat, ICELLNET, and NATMI as top three methods. These indications are available in sections “Abstract” and “Benchmarking summary” of the original manuscript.

### Liu et al. (Genome Biology, 2022)

Tested tools: CellCall (v0.0.0.9000), CellChat(v1.0.0), CellPhoneDB (v2), CellPhoneDB (v3), Connectome (v1.0.1), CytoTalk (v4.0.11), Domino (v0.1.1), Giotto (v1.0.4), ICELLNET (v0.99.3), iTALK (v0.1.0), NATMI, NicheNet (v1.0.0), scMLnet (v0.1.0), SingleCellSignalR (v1.4.0), stLearn (v0.4.7).

Tested LR resource: CellChatDB was used for all tools. For the CCC tools that do not allow to specify an input ligand-receptor database, their outputs were filtered to contain only LR pairs included in CellChatDB.

Assessment: Authors integrated scRNA-seq with ST data, from both simulated (15) and real (5) dataset. They characterize CCI into 2 main categories (i.e. short-range and long-range interactions), using spatial distance distributions between ligands and receptors, to identify near and far cell type pairs. Based on this classification, they evaluate the predicted CCI in terms of distance enrichment score (DES), a novel metric developed by the authors to measure the consistency between expected and observed spatial tendency. Besides DES, the authors also evaluate the similarity of predicted CCI across tools, identifying as ground truth the CCI interactions reported at least 3 times among 10 CCI tools, and the computing precision, recall and F1 scores.

Reported ranking: Authors provide the ranking of the tools in terms of DES, Precision, Recall, and F1-score (Figure 9 and sections “Distance enrichment score rank” and “Metrics average rank” in the original manuscript). In our work, we reported the CCC tools ranked by average DES (Figure 9C in the original manuscript), since it is the score most used by the authors to comment their results, and it was specifically developed by the authors as primary evaluation metric.

Suggested tools: Authors reported CellChat as the best performing tools, identifying as good performance tools also SingleCellSignalR, NicheNet, and ICELLNET (section “Abstract” and “Conclusion” in the original manuscript).

### Interpretation of the aggregated analysis of the four benchmarking studies

The aggregated analysis of the results of the four benchmarking studies, while informative, should be interpreted with caution.

First, the ranking does not account for absolute performance, but only relative order (e.g., the top-ranked method might have low absolute performance, yet still the best among all methods). Moreover, in many studies, the absolute performance of the methods is reported to be not high. It is also worth noting that in these studies, the authors tend to discuss the results more in terms of ranking rather than absolute performance, partly because many of the metrics used are sensitive to the specific scenario or characteristic being measured.

Second, different studies evaluate the methods in different ways, either by using different metrics or by analyzing slightly different aspects of the methods. Additionally, the studies report high variability in method performance depending on the datasets or scenarios tested, and rarely does a method consistently emerge as the best performer across the majority of datasets/scenarios. Nonetheless, the idea is that if a method ranks well in multiple studies, this suggests that its performance is likely less dependent on the specific choices made in individual studies, and instead indicates the overall accuracy and robustness of the tool.

Third, different studies may include slightly different versions of the tools, use different input parameters, or employ different ligand-receptor databases as input. Notably, this last aspect is known to have a significant impact on tool performance.

#

# **Supplementary Figures**

**
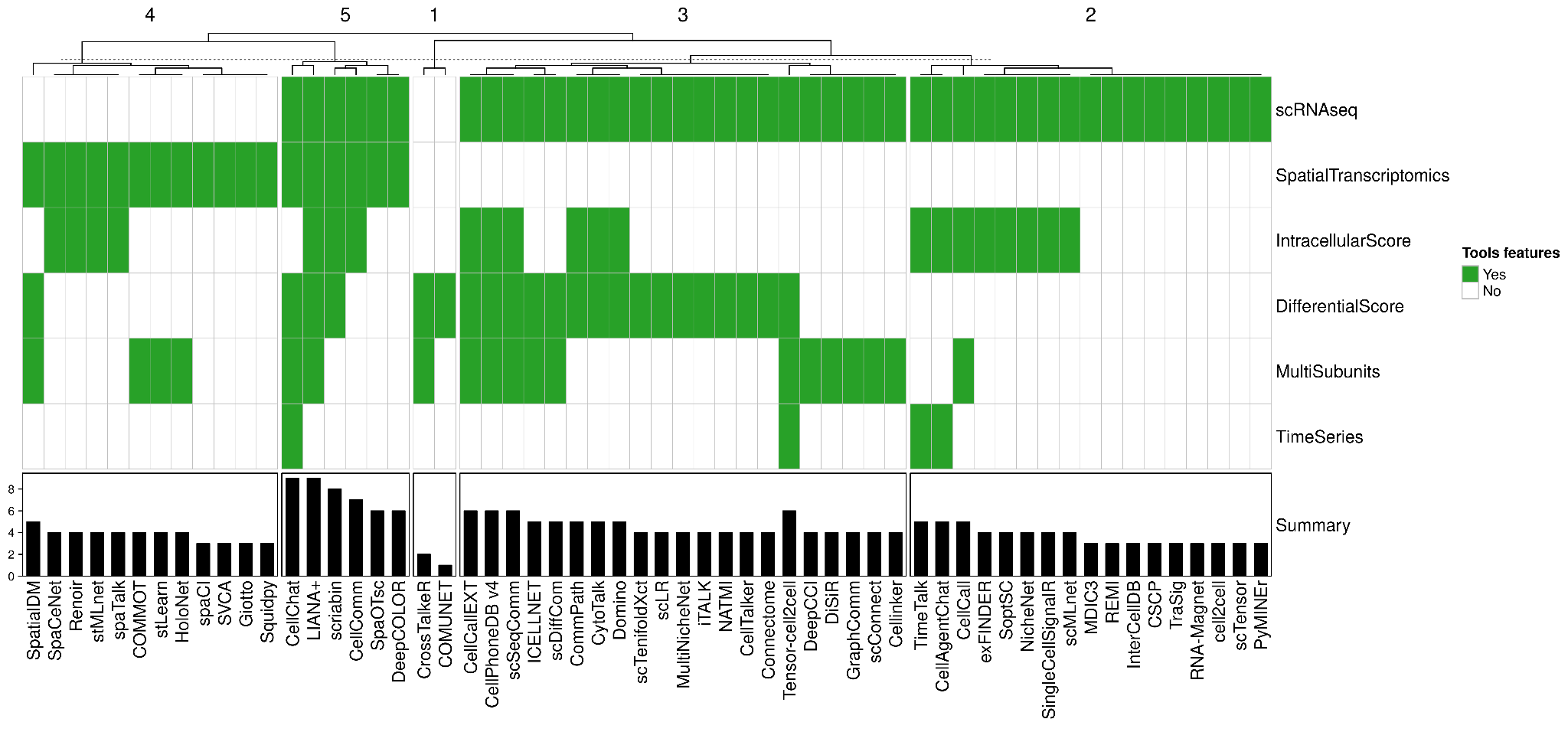
**

**Figure S1 - CCC tools clustered by their main functionalities.** The characteristics included are: capability of accepting scRNA/SpatialTranscriptomics data in input, capability of inferring an Intracellular score of the communication downstream of the receptor within the receiver cell, capability of inferring differential communication between different biological conditions, possibility to handle multi-subunits information on ligands and receptors, capability to handle datasets with series of time-points.

These characteristics were used to identify 5 groups of tools in a data-driven manner. The largest group is constituted by scRNAseq-only tools, with heterogeneous additional functionalities. In this group, 3 tools (i.e. Tensor-cell2cell, TimeTalk, CellAgentChat) emerge for the TimeSeries characteristics. Other 3 tools (i.e. CellCallEXT, CellPhoneDB v4, scSeqComm) emerge for having both IntracellularScore, DifferentialScore and MultiSubunits functionalities. The second largest group of tools comprehends the tools ST-only, with some of them capable of handling multi-subunits information. Another group comprehends the 6 tools suitable for both scRNAseq and ST data. Within this group, CellChat emerges for the DifferentialScore, MultiSubunits and TimeSeries characteristics, while scriabin and LIANA+ are characterized by intracellular and differential scoring. The Summary barplot at the bottom sums the features of the tools to provide an overview tools’ flexibility. The input features are weighted 3 points while the additional features are all weighted 1.

**
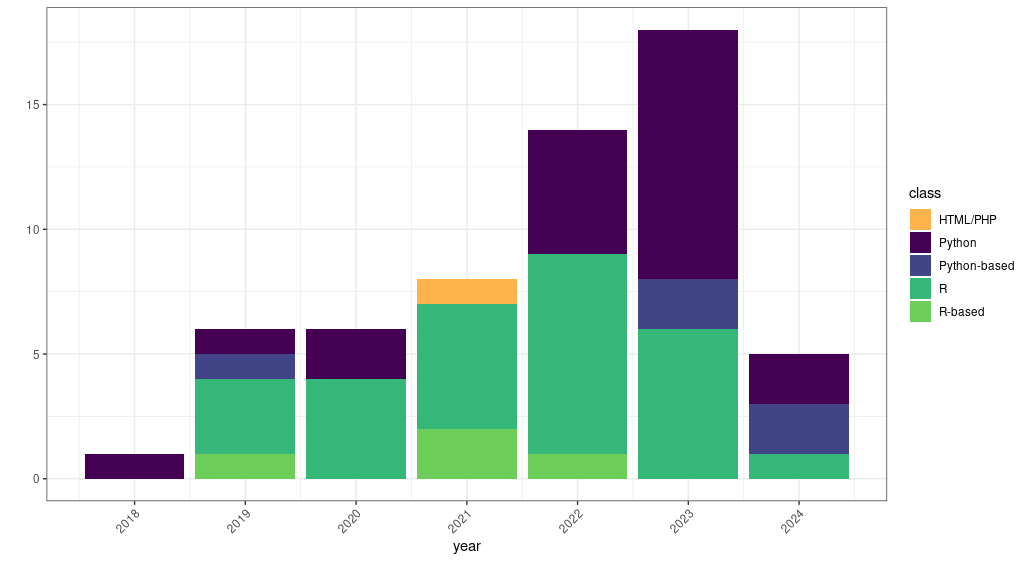
**

**Figure S2 - CCC tools programming languages.** Stacked bar plot illustrating the annual number of cell-cell communication tools published, categorized by their primary programming language (e.g., HTML, PHP, Python, R, Python-based and R-based). In this context, Python-based and R-based refer to packages primarily developed in Python and R, respectively, with support for additional languages. This visualization highlights trends in the development of CCC tools over time and the prevalence of specific programming platforms.

**
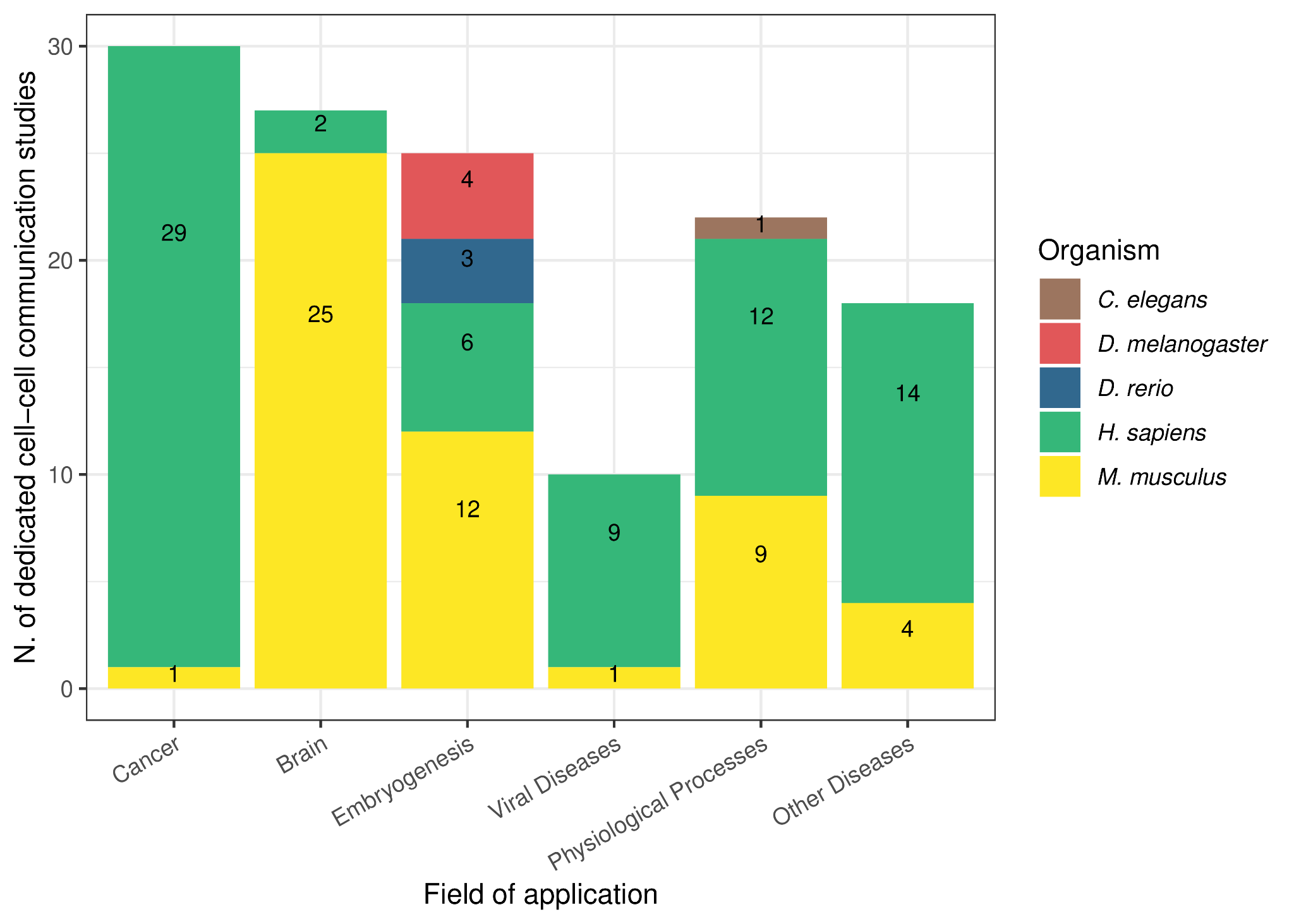
**

**Figure S3 - CCC case studies reported within CCC tools manuscripts.** Stacked bar plot illustrating the distribution of studies analyzing cell-cell communication across various research fields, represented by individual bars. The colored segments within each bar indicate the organisms studied in each field. This visualization underscores the diversity of research focus, highlighting variations in both application areas and species investigated.

#

# **Supplementary Tables**

**Table S1. CCC resources.** Main information about reviewed CCC resources, including annotated species, presence of information on intracellular signaling and protein complexes, and list of CCC tools that include the CCC resource as one of their built-in DBs. Additional information are available in Supplementary Data 1. Species: H=human, M=mouse, C=chicken, Z=zebrafish, R=rat, CH=SARS-Cov-2 Human.

| **CCC resource** | **Species** | **Intracellular interactions** | **Information about protein complexes** | **CCC Tools using the CCC resource** |
| --- | --- | --- | --- | --- |
| CellPhoneDB | H | Yes | Yes | CellPhoneDB ; Domino ; scSeqComm ; CommPath ; LIANA+ |
| Ramilowski2015 | H | No | No | CellTalker ; Connectome ; RNA-Magnet ; scSeqComm ; Giotto ; CommPath ; REMI ; TraSig |
| CellTalkDB | H ; M | No | No | CellCallEXT ; CellAgentChat LIANA+ |
| ConnectomeDB2020 | H | No | No | NATMI ; scSeqComm ; stLearn ; LIANA+ |
| ICELLNET | H | No | Yes | ICELLNET ; scSeqComm ; LIANA+ |
| GtoPdb | H ; M ; CH ; Other | No | No | scConnect ; LIANA+ |
| Cellinker-DB | H ; M ; CH | No | Yes | Cellinker ; CellCallEXT ; LIANA+ |
| CellChatDB | H ; M; Z | No | Yes | CellChat v2 ; Tensor-cell2cell ; scSeqComm ; HoloNet ; CellCallEXT ; SpatialDM ; LIANA+ |
| LRdb | H | Yes | No | SingleCellSignalR ; scSeqComm ; LIANA+ |
| Jinyu2020 | H | Yes | No | scMLnet |
| Omnipath | H ; M ; R | Yes | Yes | Squidpy ; GraphComm ; MultiNicheNet |
| iTALK-DB | H | No | No | iTALK ; scSeqComm ; LIANA+ |
| CellCall-DB | H ; M | Yes | Yes | LIANA+ ; CellCall |
| LIANA Consensus | H | Yes | Yes | scriabin ; scSeqComm ; LIANA+ |
| LRBase | H ; M ; R ; C ; Z ; other 129 organism | No | No | scTensor |
| NicheNet-DB | H ; M | Yes | No | NicheNet ; MultiNicheNet ; scSeqComm |
| Qiao2014 | H | Yes | No | scSeqComm |
| DLRP | H | No | No |  |
| CCIDB | H ; M; C | No | No |  |
| LRIDB | H ; M | No | Yes | DeepCCI |
| scDiffCom-DB | H ; M | No | Yes | scDiffCom ; scSeqComm |
| CytoTalk-DB | H | No | No | CytoTalk ; scSeqComm |
| stMLnet-DB | H | Yes | Yes | stMLnet |
| LRT-KB | H ; M | Yes | No | spaTalk ; scSeqComm |
| exFINDER-DB | H ; M ; Z | Yes | No | exFINDER |
| Zhang2019 | H | No | No | scSeqComm |

**Table S2. CCC tools - Input.** Main information about reviewed CCC tools in terms of input, including the source of input LR DB (user specified and/or built-in) and the type of input data (scRNAseq = single cell transcriptomics; STall = spatial transcriptomics (all technologies); STspot = spatial transcriptomics (spot based technology); STsc = spatial transcriptomics (single cell resolution technology); LRanalysis = output of a CCC tool; microarray = microarray data; bulk = bulk RNA-seq data). Additional information are available in Supplementary Data 2.

| **CCC Tool** | **LR DB - Source** | **LR DB - Built-in DB** | **INPUT - Type of data** |
| --- | --- | --- | --- |
| CellPhoneDB (v4.1) | built-in; user-specified | CellPhoneDB | scRNAseq |
| CellTalker | built-in; user-specified | Ramilowski2015 | scRNAseq |
| Connectome | built-in; user-specified | Ramilowski2015 | scRNAseq |
| NATMI | built-in; user-specified | ConnectomeDB2020 | scRNAseq; bulk |
| ICELLNET | built-in | ICELLNET | scRNAseq; bulk; microarray |
| scConnect | built-in | GtoPdb | scRNAseq |
| Cellinker | built-in | Cellinker-DB | scRNAseq |
| CellChat | built-in; user-specified; built-in + user-specified | CellChatDB | scRNAseq; STall |
| scDiffCom | built-in | scDiffCom-DB | scRNAseq |
| scTensor | built-in | LRBase | scRNAseq |
| Tensor-cell2cell | built-in; user-specified | CellChatDB[v1]* | scRNAseq; bulk |
| iTALK | built-in; user-specified | iTALK-DB | scRNAseq |
| PyMINEr | built-in | none | scRNAseq |
| cell2cell | built-in; user-specified | cell2cell-DB | scRNAseq; bulk |
| RNA-Magnet | built-in | Ramilowski2015* | scRNAseq |
| SingleCellSignalR | built-in | LRdb | scRNAseq |
| CytoTalk | built-in; user-specified | CytoTalk-DB | scRNAseq |
| scMLnet | built-in | Jinyu2020 | scRNAseq |
| Domino | built-in | CellPhoneDB | scRNAseq |
| CellCall | built-in | CellCall-DB | scRNAseq |
| scSeqComm | built-in; user-specified | Qiao2014; NicheNet-DB; LRdb; Choi2015; ConnectomeDB2020; CellPhoneDB; CellChatDB; Kumar2018; scDiffCom-DB; LIANA Consensus; ICELLNET; Ramilowski2015; LRT-KB; iTALK-DB; Zhang2019; Zhao2023; Cain2020; Ding2016; CytoTalk-DB; Sheikh2019; Skelly2018; Yuzwa2016 | scRNAseq |
| NicheNet | built-in | NicheNet-DB | scRNAseq; bulk |
| COMUNET | none | none | LRanalysis |
| SoptSC | user-specified | none | scRNAseq |
| Giotto | built-in; user-specified | Ramilowski2015 | STall |
| stLearn | built-in | ConnectomeDB2020 | STall |
| Squidpy | built-in | Omnipath | STall |
| stMLnet | built-in | stMLnet-DB | STall |
| HoloNet | built-in; user-specified | CellChatDB[v1] | STall |
| spaTalk | built-in; user-specified | LRT-KB | STall |
| COMMOT | user-specified | none | STall |
| CrossTalkeR | user-specified | none | LRanalysis |
| TraSig | built-in | Ramilowski2015* | scRNAseq |
| GraphComm | built-in | OmniPath | scRNAseq |
| scriabin | built-in | LIANA Consensus | scRNAseq; STspot |
| SVCA | user-specified | none | STsc |
| SpaOTsc | none | none | scRNAseq AND STsc |
| CSCP | user-specified | none | scRNAseq |
| CellCallEXT | built-in | NATMI; Cellinker-DB; CellTalkDB; CellChatDB; STRING; KEGG | scRNAseq |
| CommPath | built-in | CellPhoneDB;Ramilowski2015 | scRNAseq |
| MultiNicheNet | built-in | NicheNet-DB;Omnipath;Verschueren2020 | scRNAseq |
| scLR | user-specified | none | scRNAseq |
| scTenifoldXct | none; user-specified | none | scRNAseq |
| TimeTalk | user-specified | none | scRNAseq |
| DiSiR | user-specified | none | scRNAseq |
| InterCellDB | built-in | STRING | scRNAseq |
| CellComm | user-specified | none | scRNAseq; STall |
| exFINDER | built-in | exFINDER-DB | scRNAseq |
| DeepCOLOR | user-specified | none | scRNAseq; STall |
| Renoir | user-specified | none | STall |
| SpaCeNet | none | none | STsc |
| spaCI | none; user-specified | none | STsc |
| SpatialDM | built-in; user-specified | CellChatDB | STall |
| CellAgentChat | built-in; user-specified | CellTalkDB | scRNAseq |
| DeepCCI | built-in | LRIDB | scRNAseq |
| LIANA+ | built-in; user-specified | Baccin2020; CellCall-DB; CellChatDB; Cellinker-DB; CellPhoneDB; CellTalkDB; ConnectomeDB2020; Ramilowski2015*; GtoPdb; HPMR; ICELLNET; iTALK-DB; Kiroauc2010; LRdb; Ramilowski2015; LIANA Consensus | scRNAseq; STall |
| REMI | built-in; user-specified | Ramilowski2015* | scRNAseq; bulk |
| MDIC3 | none | none | scRNAseq |

**Table S3. CCC tools - Intercellular signaling.** Main information about reviewed CCC tools in terms of intercellular signaling inference, including whether the intercellular scoring scheme handles ligand/receptor multisubunit, what the intercellular scoring scheme is modelling/measuring (*specificity* = it prioritizes intercellular signaling based on their specificity, e.g. signaling that is exclusive to a given source-target cell clusters, or to few of them, typically ignoring the intensity of the signaling; *intensity* = it prioritizes intercellular signaling based on their intensity, e.g. strong evidence of ongoing intercellular signaling based on high expression level of ligand and receptors, typically ignoring the specificity of the signaling ; *other* = The intercellular scoring scheme prioritizes intercellular signaling based on criteria not based on intensity or specificity), and the type of intercellular score provided as output (continuous value or binary value). Additional information are available in Supplementary Data 2.

| **CCC Tool** | **Multisubunit aware** | **Intercellular score meaning** | **Intercellular score type** |
| --- | --- | --- | --- |
| CellPhoneDB (v4.1) | Yes | Specificity | Continuous |
| CellTalker | No | Intensity | Binary |
| Connectome | No | Specificity, Intensity | Continuous |
| NATMI | No | Specificity, Intensity | Continuous |
| ICELLNET | Yes | Specificity, Intensity | Continuous |
| scConnect | Yes | Specificity, Intensity | Continuous |
| Cellinker | Yes | Specificity, Intensity | Continuous |
| CellChat | Yes | Specificity | Continuous |
| scDiffCom | Yes | Specificity, Intensity | Continuous |
| scTensor | No | Intensity | Other |
| Tensor-cell2cell | Yes | Intensity, Other | Continuous |
| iTALK | No | Intensity | Binary |
| PyMINEr | No | Other | Other |
| cell2cell | No | Specificity, Intensity | Continuous |
| RNA-Magnet | No | Specificity, Intensity | Continuous |
| SingleCellSignalR | No | Intensity | Continuous |
| CytoTalk | No | Specificity, Intensity | Continuous |
| scMLnet | No | Intensity | Binary |
| Domino | No | Intensity | Continuous |
| CellCall | Yes | Intensity | Continuous |
| scSeqComm | Yes | Intensity | Continuous |
| NicheNet | No | Specificity, Intensity | Continuous |
| COMUNET | No | Intensity | Continuous |
| SoptSC | No | Intensity | Continuous |
| Giotto | No | Intensity | Continuous |
| stLearn | Yes | Intensity | Continuous |
| Squidpy | No | Intensity | Continuous |
| stMLnet | No | Intensity | Continuous |
| HoloNet | Yes | Intensity | Continuous |
| spaTalk | No | Specificity, Intensity | Continuous |
| COMMOT | Yes | Specificity, Intensity | Continuous |
| CrossTalkeR | Yes | Intensity, Other | Continuous |
| TraSig | No | Intensity | Continuous |
| GraphComm | Yes | Intensity | Continuous |
| scriabin | No | Intensity | Continuous |
| SVCA | No | Intensity | Other |
| SpaOTsc | No | Intensity | Continuous |
| CSCP | No | Intensity | Continuous |
| CellCallEXT | Yes | Specificity, Intensity | Continuous |
| CommPath | No | Intensity | Continuous |
| MultiNicheNet | No | Specificity, Intensity | Continuous |
| scLR | No | Intensity | Continuous |
| scTenifoldXct | No | Intensity | Continuous |
| TimeTalk | No | Intensity | Continuous |
| DiSiR | Yes | Specificity, Intensity | Continuous |
| InterCellDB | No | Specificity, Intensity | Continuous |
| CellComm | No | Specificity, Intensity | Continuous |
| exFINDER | No | Intensity | Continuous |
| DeepCOLOR | No | Specificity, Intensity | Other |
| Renoir | No | Specificity, Intensity | Continuous |
| SpaCeNet | No | Specificity, Intensity | Continuous |
| spaCI | No | Specificity, Intensity | Continuous |
| SpatialDM | Yes | Specificity, Intensity | Continuous |
| CellAgentChat | No | Specificity, Intensity | Continuous |
| DeepCCI | Yes | Specificity, Intensity | Continuous |
| LIANA+ | Yes | Specificity, Intensity | Other |
| REMI | No | Specificity, Intensity | Continuous |
| MDIC3 | No | Intensity | Continuous |

**Table S4. CCC tools - Intracellular signaling.** Main information about reviewed CCC tools in terms of intracellular signaling inference, including whether the CCC tools support intracellular signaling inference, whether the CCC tools use prior knowledge from public databases (e.g. signaling pathway database) during intracellular analysis [Yes] or it is only (input) data driven [No], and the presence a quantitative [Yes] or quantitative [No] output. Additional information are available in Supplementary Data 2.

| **CCC Tool** | **Support intracellular signaling analysis** | **Use of prior-knowledge during intracellular signaling analysis** | **Intracellular signaling analysis providing a quantitative output** |
| --- | --- | --- | --- |
| CellPhoneDB (v4.1) | Yes | Yes | No |
| CellTalker | No | - | - |
| Connectome | No | - | - |
| NATMI | No | - | - |
| ICELLNET | No | - | - |
| scConnect | No | - | - |
| Cellinker | No | - | - |
| CellChat | No | - | - |
| scDiffCom | No | - | - |
| scTensor | No | - | - |
| Tensor-cell2cell | No | - | - |
| iTALK | No | - | - |
| PyMINEr | No | - | - |
| cell2cell | No | - | - |
| RNA-Magnet | No | - | - |
| SingleCellSignalR | Yes | Yes | No |
| CytoTalk | Yes | No | No |
| scMLnet | Yes | Yes | No |
| Domino | Yes | Yes | No |
| CellCall | Yes | Yes | Yes |
| scSeqComm | Yes | Yes | Yes |
| NicheNet | Yes | Yes | Yes |
| COMUNET | No | - | - |
| SoptSC | Yes | Yes | No |
| Giotto | No | - | - |
| stLearn | No | - | - |
| Squidpy | No | - | - |
| stMLnet | Yes | Yes | No |
| HoloNet | No | - | - |
| spaTalk | Yes | Yes | Yes |
| COMMOT | No | - | - |
| CrossTalkeR | No | - | - |
| TraSig | No | - | - |
| GraphComm | No | - | - |
| scriabin | Yes | Yes | Yes |
| SVCA | No | - | - |
| SpaOTsc | No | - | - |
| CSCP | No | - | - |
| CellCallEXT | Yes | Yes | Yes |
| CommPath | Yes | Yes | Yes |
| MultiNicheNet | No | - | - |
| scLR | No | - | - |
| scTenifoldXct | No | - | - |
| TimeTalk | Yes | No | Yes |
| DiSiR | No | - | - |
| InterCellDB | No | - | - |
| CellComm | Yes | Yes | Yes |
| exFINDER | Yes | Yes | Yes |
| DeepCOLOR | No | - | - |
| Renoir | Yes | Yes | Yes |
| SpaCeNet | Yes | No | Yes |
| spaCI | No | - | - |
| SpatialDM | No | - | - |
| CellAgentChat | Yes | Yes | Yes |
| DeepCCI | No | - | - |
| LIANA+ | Yes | Yes | No |
| REMI | No | - | - |
| MDIC3 | No | - | - |

**Table S5. CCC tools - Differential cellular communication analysis.** Main information about reviewed CCC tools in terms of differential cellular communication analysis, including whether the CCC tools support differential cellular communication analysis, whether the tool consider intra-variability of each condition (i.e. sample/patient level) if present [Yes], or it pools all cells within an experimental condition [No], and the number of conditions that the tools are able to consider in the analysis. Additional information are available in Supplementary Data 2.

| **CCC Tool** | **Support differential cellular communication analysis** | **Capture intra-variability within a condition (if present)** | **Number of conditions considered in the analysis** |
| --- | --- | --- | --- |
| CellPhoneDB (v4.1) | Yes | No | 2 |
| CellTalker | Yes | No | 2 |
| Connectome | Yes | No | 2 |
| NATMI | Yes | No | 2 |
| ICELLNET | Yes | No | 2 |
| scConnect | No | - | - |
| Cellinker | No | - | - |
| CellChat | Yes | No | 2 |
| scDiffCom | Yes | No | 2 |
| scTensor | No | - | - |
| Tensor-cell2cell | Yes | No | unlimited |
| iTALK | Yes | No | 2 |
| PyMINEr | No | - | - |
| cell2cell | No | - | - |
| RNA-Magnet | No | - | - |
| SingleCellSignalR | No | - | - |
| CytoTalk | Yes | No | unlimited |
| scMLnet | No | - | - |
| Domino | Yes | No | 2 |
| CellCall | No | - | - |
| scSeqComm | Yes | Yes | 2 |
| NicheNet | No | - | - |
| COMUNET | Yes | No | 2 |
| SoptSC | No | - | - |
| Giotto | No | - | - |
| stLearn | No | - | - |
| Squidpy | No | - | - |
| stMLnet | No | - | - |
| HoloNet | No | - | - |
| spaTalk | No | - | - |
| COMMOT | No | - | - |
| CrossTalkeR | Yes | No | 2 |
| TraSig | No | - | - |
| GraphComm | No | - | - |
| scriabin | Yes | Yes | unlimited |
| SVCA | No | - | - |
| SpaOTsc | No | - | - |
| CSCP | No | - | - |
| CellCallEXT | Yes | No | 2 |
| CommPath | Yes | No | 2 |
| MultiNicheNet | Yes | Yes | 3 |
| scLR | Yes | Yes | 2 |
| scTenifoldXct | Yes | No | 2 |
| TimeTalk | No | - | - |
| DiSiR | No | - | - |
| InterCellDB | No | - | - |
| CellComm | No | - | - |
| exFINDER | No | - | - |
| DeepCOLOR | No | - | - |
| Renoir | No | - | - |
| SpaCeNet | No | - | - |
| spaCI | No | - | - |
| SpatialDM | Yes | Yes | 2 |
| CellAgentChat | No | - | - |
| DeepCCI | No | - | - |
| LIANA+ | Yes | Yes | unlimited |
| REMI | No | - | - |
| MDIC3 | No | - | - |

**Table S6. CCC tools - Main output and built-in visualizations.** Main information about reviewed CCC tools in terms of analysis’ output and type of built-in visualizations. Type of output provided by the CCC tool is classified in: Scores = Any kind of numerical output (score, pvalue, etc.) calculated for each LR pair and cell-type pairs or for each cell-type pairs; TensorFactorization = Results of matrix-factorization based methods, it can be the tensor loadings; Network = A graph of the inferred communication network, it can be cell-level, gene-level, multi-layer, etc.; GenesList = A list of genes involved in the cell-cell communication; CellLevelInformation = Information at cell-level about the inferred communication, not encoded by numbers; LRsList = A list of actually communicating ligand-receptor pairs. Built-in visualizations provided by the CCC tools are classified in: CircosPlot = circos plot, circle plot, chord diagram, edge bundling plot; DotPlot = dot plot, balloon plot, bubble plot, scatter plot; Heatmap = heatmap; SankeyPlot = sankey plot, river plot, waterfall plot; NetworkPlot = network plot, signalling pathways plot; SpatialPlot = plots involving cell/spot spatial coordinates; BoxPlot = boxplot; ViolinBoxPlot = violin plot; BarPlot = bar plot; WebApp = interactive webapp providing multiple visualization options. Additional information are available in Supplementary Data 2.

| **CCC Tool** | **Analysis output** | **Built-in visualizations** |
| --- | --- | --- |
| CellPhoneDB (v4.1) | Scores |  |
| CellTalker | LRsList | CircosPlot; ViolinBoxPlot |
| Connectome | Scores | Heatmap; CircosPlot; NetworkPlot |
| NATMI | Scores | Heatmap; NetworkPlot; CircosPlot |
| ICELLNET | Scores | NetworkPlot; BarPlot; DotPlot; Heatmap |
| scConnect | Scores | WebApp; DotPlot; Heatmap |
| Cellinker | Scores | WebApp |
| CellChat | Scores | NetworkPlot; CircosPlot; Heatmap; DotPlot; SankeyPlot; SpatialPlot; WebApp |
| scDiffCom | Scores | DotPlot; NetworkPlot; DotPlot |
| scTensor | TensorFactorization | Heatmap; NetworkPlot; ViolinBoxPlot |
| Tensor-cell2cell | TensorFactorization | BarPlot; Heatmap |
| iTALK | LRsList | NetworkPlot; CircosPlot; BarPlot |
| PyMINEr | Network | NetworkPlot; Heatmap |
| cell2cell | Scores | Heatmap; CircosPlot; DotPlot |
| RNA-Magnet | Scores |  |
| SingleCellSignalR | Scores | CircosPlot; Heatmap; NetworkPlot |
| CytoTalk | Network | Heatmap; NetworkPlot |
| scMLnet | Network | NetworkPlot; Heatmap |
| Domino | Network | Heatmap; NetworkPlot |
| CellCall | Scores | CircosPlot; Heatmap; SankeyPlot; SankeyPlot |
| scSeqComm | Scores | CircosPlot; Heatmap; NetworkPlot |
| NicheNet | Scores | Heatmap; CircosPlot |
| COMUNET | LRsList | NetworkPlot |
| SoptSC | Scores | CircosPlot |
| Giotto | Scores | WebApp; Heatmap; DotPlot; BarPlot; NetworkPlot |
| stLearn | Scores | Heatmap; ViolinBoxPlot; BarPlot |
| Squidpy | Scores | DotPlot |
| stMLnet | Scores; Network | CircosPlot; NetworkPlot; Heatmap; SankeyPlot; |
| HoloNet | Scores; Network | NetworkPlot; Heatmap |
| spaTalk | Scores; Network | Heatmap; SankeyPlot; SpatialPlot; NetworkPlot |
| COMMOT | Scores | NetworkPlot; Heatmap |
| CrossTalkeR | Network | NetworkPlot |
| TraSig | Network; CellLevelInformation | Heatmap; NetworkPlot |
| GraphComm | Scores | NetworkPlot; BarPlot |
| scriabin | Scores; Network | SankeyPlot; DotPlot; Heatmap; CircosPlot |
| SVCA | Scores | SankeyPlot; ViolinBoxPlot |
| SpaOTsc | Scores |  |
| CSCP | CellLevelInformation |  |
| CellCallEXT | Scores | DotPlot; SankeyPlot; CircosPlot; Heatmap |
| CommPath | Scores | CircosPlot; Heatmap; DotPlot; NetworkPlot |
| MultiNicheNet | LRsList | DotPlot; CircosPlot; NetworkPlot |
| scLR | Scores | Heatmap |
| scTenifoldXct | Scores | NetworkPlot |
| TimeTalk | Scores |  |
| DiSiR | Scores | NetworkPlot; Heatmap; CircosPlot |
| InterCellDB | Scores | DotPlot; BarPlot; SpatialPlot |
| CellComm | Scores | DotPlot; NetworkPlot; BarPlot; Heatmap |
| exFINDER | Scores | NetworkPlot; CircosPlot; BarPlot; NetworkPlot |
| DeepCOLOR | Scores | SpatialPlot |
| Renoir | Scores | Heatmap; SankeyPlot |
| SpaCeNet | Scores; Network |  |
| spaCI | Scores |  |
| SpatialDM | Scores | Heatmap; DotPlot |
| CellAgentChat | Scores | WebApp |
| DeepCCI | Scores | Heatmap; NetworkPlot; DotPlot; CircosPlot |
| LIANA+ | Scores; TensorFactorization; GenesList | DotPlot |
| REMI | Scores | CircosPlot; Heatmap; ViolinBoxPlot; BarPlot |
| MDIC3 | Scores | Heatmap |

**Table S7. CCC tools - Software.** Main information about reviewed CCC tools in terms of software implementation, including the programming language, the release format, the support to parallel execution, and the additional indicators of software quality. Release format: *RPackage* = released as R package; *PythonPackageLibrary* = released as Python package library; *PythonPackageCmdLine* = released as Python package command line only; *RScript* = released as a set of R scripts; *PythonScript* = released as a set of Python scripts; *Matlab* = released as a set Matlab scripts; *webServer* = online web server hosting the application; *webAPI*: online web API; *webApp*: local web app. Software quality: *tutorial* = availability of any kind of tutorial, vignette, hands on, examples, ... that shows how to use the software in a practical way; *documentation* = availability of documentation of CCC tool's functions, input parameters, and functionalities; *codeDocumentation* = availability of documentation (i.e. comments) in the source code; *installationInstruction* = availability of installation instructions (including required dependencies, if any); *testDataset* = availability of (or instruction to retrieve) at least one dataset to test the CCC tool; *container* = presence of a container (e.g. Docker, Singularity) including the CCC tool. Additional information are available in Supplementary Data 2.

| **CCC Tool** | **Programming language** | **Release format** | **Support parallelism** | **Software quality features** |
| --- | --- | --- | --- | --- |
| CellPhoneDB (v4.1) | Python | PythonPackageLibrary | Yes | tutorial; codeDocumentation; installationInstruction; testDataset |
| CellTalker | R | RPackage | No | tutorial; codeDocumentation; installationInstruction; testDataset |
| Connectome | R | RPackage | No | tutorial; documentation; codeDocumentation; installationInstruction; testDataset |
| NATMI | Python | PythonPackageCmdLine | Yes | tutorial; documentation; codeDocumentation; installationInstruction; testDataset; container |
| ICELLNET | R | RPackage | No | tutorial; documentation; codeDocumentation; installationInstruction; testDataset |
| scConnect | Python | PythonPackageLibrary | No | tutorial; documentation; codeDocumentation; installationInstruction; testDataset |
| Cellinker | HTML; PHP | webServer; webAPI | No | tutorial; documentation; testDataset |
| CellChat | R; C++ | RPackage | Yes | tutorial; documentation; codeDocumentation; installationInstruction; testDataset |
| scDiffCom | R | RPackage | Yes | tutorial; documentation; codeDocumentation; installationInstruction; testDataset |
| scTensor | R | RPackage | No | tutorial; documentation; codeDocumentation; installationInstruction; testDataset |
| Tensor-cell2cell | Python | PythonPackageLibrary | No | tutorial; documentation; codeDocumentation; installationInstruction; testDataset |
| iTALK | R | RPackage | Yes | tutorial; documentation; codeDocumentation; installationInstruction; testDataset |
| PyMINEr | Python | PythonPackageLibrary; PythonPackageCmdLine | No | tutorial; documentation; codeDocumentation; installationInstruction; testDataset |
| cell2cell | Python | PythonPackageLibrary | Yes | tutorial; documentation; codeDocumentation; installationInstruction; testDataset |
| RNA-Magnet | R | RPackage | No | tutorial; documentation; codeDocumentation; installationInstruction; testDataset |
| SingleCellSignalR | R | RPackage | No | tutorial; documentation; codeDocumentation; installationInstruction; testDataset |
| CytoTalk | R; Matlab | RPackage; Matlab | Yes | tutorial; documentation; codeDocumentation; installationInstruction; testDataset |
| scMLnet | R | RPackage | No | tutorial; documentation; codeDocumentation; installationInstruction; testDataset |
| Domino | R | RPackage | No | tutorial; documentation; codeDocumentation; installationInstruction; testDataset |
| CellCall | R | RPackage | No | tutorial; documentation; codeDocumentation; installationInstruction; testDataset |
| scSeqComm | R; C++ | RPackage | Yes | tutorial; documentation; codeDocumentation; installationInstruction; testDataset |
| NicheNet | R | RPackage | No | tutorial; documentation; codeDocumentation; installationInstruction; testDataset |
| COMUNET | R | RPackage | No | tutorial; documentation; codeDocumentation; installationInstruction; testDataset |
| SoptSC | R; Matlab | RPackage; Matlab | Yes | tutorial; documentation; codeDocumentation; installationInstruction; testDataset |
| Giotto | R | RPackage | Yes | tutorial; documentation; codeDocumentation; installationInstruction; testDataset |
| stLearn | Python | PythonPackageLibrary; WebApp | Yes | tutorial; documentation; codeDocumentation; installationInstruction; testDataset |
| Squidpy | Python | PythonPackageLibrary | Yes | tutorial; documentation; codeDocumentation; installationInstruction; testDataset |
| stMLnet | R | RPackage | Yes | tutorial; documentation; codeDocumentation; installationInstruction; testDataset |
| HoloNet | Python | PythonPackageLibrary | No | tutorial; documentation; codeDocumentation; installationInstruction; testDataset |
| spaTalk | R | RPackage | Yes | tutorial; documentation; codeDocumentation; installationInstruction; testDataset |
| COMMOT | Python | PythonPackageLibrary | No | tutorial; documentation; codeDocumentation; installationInstruction; testDataset |
| CrossTalkeR | R | RPackage | No | tutorial; documentation; codeDocumentation; installationInstruction; testDataset |
| TraSig | Python | PythonPackageCmdLine | Yes | tutorial; documentation; codeDocumentation; installationInstruction; testDataset |
| GraphComm | Python | PythonScript | No | tutorial; testDataset |
| scriabin | R | RPackage | No | tutorial; documentation; codeDocumentation; installationInstruction; testDataset |
| SVCA | R; Python | PythonPackageLibrary; PythonPackageCmdLine | No | tutorial; installationInstruction; testDataset |
| SpaOTsc | Python | PythonPackageLibrary | No | tutorial; documentation; codeDocumentation; installationInstruction; testDataset |
| CSCP | Python | PythonScript | No | installationInstruction; testDataset |
| CellCallEXT | R | RPackage | No | tutorial; documentation; codeDocumentation; installationInstruction; testDataset |
| CommPath | R | RPackage; WebServer | No | tutorial; documentation; codeDocumentation; installationInstruction; testDataset |
| MultiNicheNet | R | RPackage | Yes | tutorial; documentation; codeDocumentation; installationInstruction; testDataset |
| scLR | R | RPackage | Yes | tutorial; documentation; codeDocumentation; testDataset |
| scTenifoldXct | Python | PythonPackageLibrary; WebServer | Yes | tutorial; documentation; codeDocumentation; installationInstruction; testDataset; container |
| TimeTalk | R | RPackage | Yes | tutorial; documentation; codeDocumentation; installationInstruction; testDataset |
| DiSiR | R; Python | PythonPackageCmdLine | No | tutorial; documentation; codeDocumentation; installationInstruction; testDataset |
| InterCellDB | R | RPackage | Yes | tutorial; documentation; codeDocumentation; installationInstruction; testDataset |
| CellComm | R | RPackage | Yes | tutorial; documentation; codeDocumentation; installationInstruction; testDataset |
| exFINDER | R | RPackage | No | tutorial; documentation; codeDocumentation; installationInstruction; testDataset |
| DeepCOLOR | Python | PythonPackageLibrary | No | tutorial; installationInstruction; testDataset |
| Renoir | Python | PythonPackageLibrary | Yes | tutorial; documentation; codeDocumentation; installationInstruction; testDataset |
| SpaCeNet | Python | PythonPackageLibrary; PythonPackageCmdLine | Yes | tutorial; documentation; codeDocumentation; installationInstruction; testDataset; container |
| spaCI | Python | PythonPackageLibrary; PythonPackageCmdLine | No | tutorial; documentation; installationInstruction; testDataset |
| SpatialDM | Python | PythonPackageLibrary | No | tutorial; documentation; codeDocumentation; installationInstruction; testDataset |
| CellAgentChat | R; Python | PythonPackageLibrary | No | tutorial; documentation; installationInstruction; testDataset |
| DeepCCI | R; Python | PythonPackageCmdLine | Yes | tutorial; installationInstruction; testDataset |
| LIANA+ | Python | PythonPackageLibrary | Yes | tutorial; documentation; codeDocumentation; installationInstruction; testDataset |
| REMI | R | RPackage | No | tutorial; documentation; codeDocumentation; installationInstruction; testDataset |
| MDIC3 | R; Python | PythonPackageCmdLine | Yes | tutorial; installationInstruction; testDataset |

**Table S8. CCC tools - Assessment and validation.** Main information about reviewed CCC tools in terms of their assessment and validation in the original manuscript, including the list of CC tools compared with the given CCC tool (if any), the type of validation adopted to assess the tool, and the presence of a computational burden assessment. Type of validation adopted to assess/validate the CCC tool: *Direct validation* = experimental validation of predicted intercellular and/or intracellular signaling, typically involving few of the CCC tools predictions (e.g. immunostaining, RNAscope, qPCR); *Indirect validation* = use of complementary data (e.g. spatial transcriptomics, proteomics...) or use of dataset in controlled environment (e.g. knockout experiments, perturbation seq, or dataset where the biological "outcome" is known); *Literature agreement* = comparison of the predicted intercellular and/or intracellular signaling with current biological/medical literature; *Robustness* = test stability of the tool to noise (e.g. subsampling, dropping out, cell clustering); *Simulation data* = use of simulated gene expression data with known ongoing intercellular and/or intracellular signaling. Additional information are available in Supplementary Data 2.

| **CCC Tool** | **Comparison with other tools**  **(if any)** | **Type of validation**  **(if any)** | **Computational burden assessment** |
| --- | --- | --- | --- |
| CellPhoneDB (v4.1) |  | Literature agreement; Direct validation | Yes |
| CellTalker |  | Literature agreement; Direct validation | No |
| Connectome |  | Literature agreement | Yes |
| NATMI | CellPhoneDB | Literature agreement | Yes |
| ICELLNET |  | Literature agreement; Direct validation; Robustness; Indirect validation | No |
| scConnect | CellPhoneDB | Literature agreement; Robustness | No |
| Cellinker |  |  | No |
| CellChat | SingleCellSignalR; iTALK; CellPhoneDB | Literature agreement; Direct validation; Robustness | Yes |
| scDiffCom |  | Literature agreement; Indirect validation | No |
| scTensor | CellPhoneDB, Giotto, CrossTalkeR, Squidpy, NATMI, FunRes, ICELLNET, TraSig, Halpern’s score, SingleCellSignalR, CellTalkDB | Literature agreement; Simulation data | Yes |
| Tensor-cell2cell |  | Literature agreement; Robustness; Simulation data | Yes |
| iTALK |  |  | No |
| PyMINEr | No | Literature agreement; Direct validation | No |
| cell2cell | CellChat, ICELLNET, “Smille” score, LR counts | Literature agreement; Direct Validation; Indirect validation | No |
| RNA-Magnet |  | Literature agreement; Direct validation; Indirect validation | No |
| SingleCellSignalR | product score, average score, pvalues, Zhou | Literature agreement; Direct validation; Indirect validation | No |
| CytoTalk | Skelly, Kumar, Zhou, NicheNet, CellPhoneDB, SoptSC, | Literature agreement; Indirect validation | No |
| scMLnet | NicheNet, CCCExplorer | Literature agreement; Indirect validation; Direct validation; Robustness | No |
| Domino |  | Literature agreement; Direct validation | No |
| CellCall | CellPhoneDB, CellChat, iTALK, SingleCellSignalR | Literature agreement; Direct validation | No |
| scSeqComm | Zhou, Skelly, SingleCellSignalR | Literature agreement; Indirect validation | No |
| NicheNet | Ingenuity Pathway Analysis, CCCExplorer | Literature agreement; Indirect validation | No |
| COMUNET |  | Literature agreement | No |
| SoptSC |  | Literature agreement | No |
| Giotto |  | Literature agreement; Simulation data | No |
| stLearn | Squidy, CellPhoneDB, NATMI, SingleCellSignalR, CellChat, NCEM, SpaTalk, SpaOTsc | Literature agreement; Direct validation; Robustness; Simulation data | No |
| Squidpy |  | Literature agreement | Yes |
| stMLnet | NicheNet, CytoTalk, MISTy | Literature agreement; Simulation data; Indirect validation | No |
| HoloNet | NicheNet, SpaTalk | Literature agreement; Robustness; Simulation data | No |
| spaTalk | RCTD, Seurat, SPOTlight, deconvSeq, Stereoscope, cell2localization, Giotto, SpaOTsc, NicheNet, CytoTalk, CellChat, CellPhoneDB, CellCall | Literature agreement | Yes |
| COMMOT | CellPhoneDB Giotto, CellChat | Literature agreement; Direct Validation; Robustness | Yes |
| CrossTalkeR |  | Literature agreement | No |
| TraSig | CellPhoneDB SingleCellSignalR | Literature agreement; Direct validation | No |
| GraphComm |  | Literature agreement; Indirect validation | No |
| scriabin | NATMI, CellChat, iTALK, Connectome, SCA | Literature agreement; Robustness; Indirect validation | Yes |
| SVCA |  | Literature agreement; Simulation data | No |
| SpaOTsc |  | Literature agreement; Robustness | No |
| CSCP | CellCall, CellChat, CellPhoneDB, CytoTalk, ICELLNET, Kumar, NATMI, NicheNet, SingleCellSignalR, Skelly, iTALK, scConnect, scMLnet | Literature agreement; Indirect validation | No |
| CellCallEXT | NicheNet | Literature agreement | No |
| CommPath |  | Literature agreement | No |
| MultiNicheNet |  | Literature agreement; Indirect validation | No |
| scLR | CellChat, iTALK | Literature agreement; Simulation data | No |
| scTenifoldXct | CellChat, Connectome, iTALK, NATMI, SingleCellSignalR | Literature agreement; Indirect validation | No |
| TimeTalk |  | Literature agreement | No |
| DiSiR | CellPhoneDB, ICELLNET | Literature agreement; Simulation data | No |
| InterCellDB | CellChat, iTALK, CellPhoneDB, SingleCellSignalR, NicheNet | Literature agreement; | Yes |
| CellComm | NicheNet, SoptSC, CytoTalk, CellPhoneDB | Direct validation | No |
| exFINDER | CellChat, ICELLNET | Literature agreement | No |
| DeepCOLOR |  | Literature agreement; Direct validation; Robustness; Simulation data | No |
| Renoir |  | Literature agreement | No |
| SpaCeNet | MISTy | Literature agreement; Simulation data | Yes |
| spaCI | iTALK, CellPhoneDB, CellChat, Connectome | Literature agreement; Simulation data | No |
| SpatialDM | CellChat, SpaTalk, SpatialCorr, Giotto | Literature agreement; Simulation data | Yes |
| CellAgentChat | CellPhoneDB (v5), CellChat (v2), NICHES, COMMOT, Scriabin | Literature agreement; Simulation data | No |
| DeepCCI | SingleCellSignalR, iTALK, CellPhoneDB, CellChat, CellCall, CytoTalk, NATMI | Literature agreement; Indirect validation | Yes |
| LIANA+ |  | Literature agreement; Indirect validation | No |
| REMI | NicheNet, NATMI, CellPhoneDB, CCCExplorer | Direct validation; Robustness; Simulation data | No |
| MDIC3 | CellChat, iTALK, CellPhoneDB | Literature agreement | Yes |

# **Supplementary References**

1. Kumar MP, Du J, Lagoudas G, et al. Analysis of Single-Cell RNA-Seq Identifies Cell-Cell Communication Associated with Tumor Characteristics. Cell Rep. 2018; 25:1458-1468.e4

2. Baccin C, Al-Sabah J, Velten L, et al. Combined single-cell and spatial transcriptomics reveal the molecular, cellular and spatial bone marrow niche organization. Nat. Cell Biol. 2020; 22:38–48

3. Liu Y, Li JSS, Rodiger J, et al. FlyPhoneDB: an integrated web-based resource for cell–cell communication prediction in Drosophila. Genetics 2022; 220:iyab235

4. Xu C, Ma D, Ding Q, et al. PlantPhoneDB: A manually curated pan-plant database of ligand-receptor pairs infers cell–cell communication. Plant Biotechnol. J. 2022; 20:2123–2134

5. Armingol E, Ghaddar A, Joshi CJ, et al. Inferring a spatial code of cell-cell interactions across a whole animal body. PLOS Comput. Biol. 2022; 18:e1010715

6. Raredon MSB, Adams TS, Suhail Y, et al. Single-cell connectomic analysis of adult mammalian lungs. Sci. Adv. 2019; 5:eaaw3851

7. Zheng R, Zhang Y, Tsuji T, et al. MEBOCOST: Metabolite-mediated Cell Communication Modeling by Single Cell Transcriptome. 2022; 2022.05.30.494067

8. Kirouac DC, Ito C, Csaszar E, et al. Dynamic interaction networks in a hierarchically organized tissue. Mol. Syst. Biol. 2010; 6:417

9. Zhao W, Johnston KG, Ren H, et al. Inferring neuron-neuron communications from single-cell transcriptomics through NeuronChat. Nat. Commun. 2023; 14:1128

10. Ding C, Li Y, Guo F, et al. A Cell-type-resolved Liver Proteome*. Mol. Cell. Proteomics 2016; 15:3190–3202

11. Cain MP, Hernandez BJ, Chen J. Quantitative single-cell interactomes in normal and virus-infected mouse lungs. Dis. Model. Mech. 2020; 13:dmm044404

12. Sheikh BN, Bondareva O, Guhathakurta S, et al. Systematic Identification of Cell-Cell Communication Networks in the Developing Brain. iScience 2019; 21:273–287

13. Yuzwa SA, Yang G, Borrett MJ, et al. Proneurogenic Ligands Defined by Modeling Developing Cortex Growth Factor Communication Networks. Neuron 2016; 91:988–1004

14. Skelly DA, Squiers GT, McLellan MA, et al. Single-Cell Transcriptional Profiling Reveals Cellular Diversity and Intercommunication in the Mouse Heart. Cell Rep. 2018; 22:600–610

15. Pavličev M, Wagner GP, Chavan AR, et al. Single-cell transcriptomics of the human placenta: inferring the cell communication network of the maternal-fetal interface. Genome Res. 2017; 27:349–361

16. Choi H, Sheng J, Gao D, et al. Transcriptome Analysis of Individual Stromal Cell Populations Identifies Stroma-Tumor Crosstalk in Mouse Lung Cancer Model. Cell Rep. 2015; 10:1187–1201

17. Choi H, Sheng J, Gao D, et al. Transcriptome Analysis of Individual Stromal Cell Populations Identifies Stroma-Tumor Crosstalk in Mouse Lung Cancer Model. Cell Rep. 2015; 10:1187–1201

18. Wang K, Patkar S, Lee JS, et al. Deconvolving Clinically Relevant Cellular Immune Cross-talk from Bulk Gene Expression Using CODEFACS and LIRICS Stratifies Patients with Melanoma to Anti–PD-1 Therapy. Cancer Discov. 2022; 12:1088–1105

19. Villemin J-P, Bassaganyas L, Pourquier D, et al. Inferring ligand-receptor cellular networks from bulk and spatial transcriptomic datasets with BulkSignalR. Nucleic Acids Res. 2023; 51:4726–4744

20. Santvoort M van, Lapuente-Santana Ó, Finotello F, et al. Mathematically mapping the network of cells in the tumor microenvironment. 2023; 2023.02.03.526946

21. Tran M, Yoon S, Teoh M, et al. A robust experimental and computational analysis framework at multiple resolutions, modalities and coverages. Front. Immunol. 2022; 13:

22. Mishra V, Re DB, Le Verche V, et al. Systematic elucidation of neuron-astrocyte interaction in models of amyotrophic lateral sclerosis using multi-modal integrated bioinformatics workflow. Nat. Commun. 2020; 11:5579

23. Interlandi M, Kerl K, Dugas M. InterCellar enables interactive analysis and exploration of cell−cell communication in single-cell transcriptomic data. Commun. Biol. 2022; 5:1–13

24. Moratalla-Navarro F, Moreno V, Sanz-Pamplona R. TALKIEN: crossTALK IntEraction Network. A web-based tool for deciphering molecular communication through ligand–receptor interactions. Mol. Omics 2023; 19:688–696

25. Guilliams M, Bonnardel J, Haest B, et al. Spatial proteogenomics reveals distinct and evolutionarily conserved hepatic macrophage niches. Cell 2022; 185:379-396.e38

26. Browaeys R, Gilis J, Sang-Aram C, et al. MultiNicheNet: a flexible framework for differential cell-cell communication analysis from multi-sample multi-condition single-cell transcriptomics data. 2023; 2023.06.13.544751

27. Xin Y, Lyu P, Jiang J, et al. LRLoop: a method to predict feedback loops in cell–cell communication. Bioinformatics 2022; 38:4117–4126

28. Zhao W, Johnston KG, Ren H, et al. Inferring neuron-neuron communications from single-cell transcriptomics through NeuronChat. Nat. Commun. 2023; 14:1128

29. Zheng R, Zhang Y, Tsuji T, et al. MEBOCOST: Metabolite-mediated Cell Communication Modeling by Single Cell Transcriptome. 2022; 2022.05.30.494067

30. Yuan Y, Bar-Joseph Z. GCNG: graph convolutional networks for inferring gene interaction from spatial transcriptomics data. Genome Biol. 2020; 21:300

31. Li D, Ding J, Bar-Joseph Z. Identifying signaling genes in spatial single-cell expression data. Bioinformatics 2021; 37:968–975

32. Peng L, Gao P, Xiong W, et al. Identifying potential ligand–receptor interactions based on gradient boosted neural network and interpretable boosting machine for intercellular communication analysis. Comput. Biol. Med. 2024; 171:108110

33. Subedi S, Park YP. Single-cell pair-wise relationships untangled by composite embedding model. iScience 2023; 26:106025

34. Ximerakis M, Lipnick SL, Innes BT, et al. Single-cell transcriptomic profiling of the aging mouse brain. Nat. Neurosci. 2019; 22:1696–1708

35. Ru B, Huang J, Zhang Y, et al. Estimation of cell lineages in tumors from spatial transcriptomics data. Nat. Commun. 2023; 14:568

36. Bafna M, Li H, Zhang X. CLARIFY: cell–cell interaction and gene regulatory network refinement from spatially resolved transcriptomics. Bioinformatics 2023; 39:i484–i493

37. Tanevski J, Flores ROR, Gabor A, et al. Explainable multiview framework for dissecting spatial relationships from highly multiplexed data. Genome Biol. 2022; 23:97

38. Raredon MSB, Yang J, Kothapalli N, et al. Comprehensive visualization of cell–cell interactions in single-cell and spatial transcriptomics with NICHES. Bioinformatics 2023; 39:btac775

39. Fischer DS, Schaar AC, Theis FJ. Learning cell communication from spatial graphs of cells. 2021; 2021.07.11.451750

40. Tsuchiya T, Hori H, Ozaki H. CCPLS reveals cell-type-specific spatial dependence of transcriptomes in single cells. Bioinformatics 2022; 38:4868–4877

41. Jerby-Arnon L, Regev A. DIALOGUE maps multicellular programs in tissue from single-cell or spatial transcriptomics data. Nat. Biotechnol. 2022; 40:1467–1477

42. Ramirez Flores RO, Lanzer JD, Dimitrov D, et al. Multicellular factor analysis of single-cell data for a tissue-centric understanding of disease. eLife 2023; 12:e93161

43. Li H, Zhang Z, Squires M, et al. scMultiSim: simulation of multi-modality single cell data guided by cell-cell interactions and gene regulatory networks. 2023; 2022.10.15.512320

44. Mitchel J, Gordon MG, Perez RK, et al. Coordinated, multicellular patterns of transcriptional variation that stratify patient cohorts are revealed by tensor decomposition. Nat. Biotechnol. 2024; 1–10

45. Jung S, Singh K, del Sol A. FunRes: resolving tissue-specific functional cell states based on a cell–cell communication network model. Brief. Bioinform. 2021; 22:bbaa283

46. Kim H, Kumar A, Lövkvist C, et al. CellNeighborEX: deciphering neighbor‐dependent gene expression from spatial transcriptomics data. Mol. Syst. Biol. 2023; 19:e11670

47. Ren X, Zhong G, Zhang Q, et al. Reconstruction of cell spatial organization from single-cell RNA sequencing data based on ligand-receptor mediated self-assembly. Cell Res. 2020; 30:763–778

48. Li R, Yang X. De novo reconstruction of cell interaction landscapes from single-cell spatial transcriptome data with DeepLinc. Genome Biol. 2022; 23:124

49. Ghaddar B, De S. Reconstructing physical cell interaction networks from single-cell data using Neighbor-seq. Nucleic Acids Res. 2022; 50:e82

50. Wang J, Li S, Chen L, et al. SPROUT: spectral sparsification helps restore the spatial structure at single-cell resolution. NAR Genomics Bioinforma. 2022; 4:lqac069

51. Qu F, Li W, Xu J, et al. Three-dimensional molecular architecture of mouse organogenesis. Nat. Commun. 2023; 14:4599

52. Peng L, Tan J, Xiong W, et al. Deciphering ligand–receptor-mediated intercellular communication based on ensemble deep learning and the joint scoring strategy from single-cell transcriptomic data. Comput. Biol. Med. 2023; 163:107137

53. Peng L, Yuan R, Han C, et al. CellEnBoost: A Boosting-Based Ligand-Receptor Interaction Identification Model for Cell-to-Cell Communication Inference. IEEE Trans. NanoBioscience 2023; 22:705–715

54. Peng L, Xiong W, Han C, et al. CellDialog: A Computational Framework for Ligand-Receptor-Mediated Cell-Cell Communication Analysis. IEEE J. Biomed. Health Inform. 2024; 28:580–591

55. Yuan Y, Jr CC, Adams TS, et al. CINS: Cell Interaction Network inference from Single cell expression data. PLOS Comput. Biol. 2022; 18:e1010468

56. Wu D, Gaskins JT, Sekula M, et al. Inferring Cell–Cell Communications from Spatially Resolved Transcriptomics Data Using a Bayesian Tweedie Model. Genes 2023; 14:1368

57. Pancheva A, Wheadon H, Rogers S, et al. Using topic modeling to detect cellular crosstalk in scRNA-seq. PLOS Comput. Biol. 2022; 18:e1009975

58. Burdziak C, Alonso-Curbelo D, Walle T, et al. Epigenetic plasticity cooperates with cell-cell interactions to direct pancreatic tumorigenesis. Science 2023; 380:eadd5327

59. Liu S, Zhang Y, Peng J, et al. An improved hierarchical variational autoencoder for cell–cell communication estimation using single-cell RNA-seq data. Brief. Funct. Genomics 2024; 23:118–127

60. Lu M, Sha Y, Silva TC, et al. LR Hunting: A Random Forest Based Cell–Cell Interaction Discovery Method for Single-Cell Gene Expression Data. Front. Genet. 2021; 12:

61. Luecken M, Gigante S, Burkhardt D, et al. Defining and benchmarking open problems in single-cell analysis. 2024;
